# Regional distribution of unbound eletriptan and sumatriptan in the CNS and PNS in rats: implications for a potential central action

**DOI:** 10.1186/s10194-024-01894-0

**Published:** 2024-10-30

**Authors:** Nana Svane, Frida Bällgren, Aghavni Ginosyan, Mie Kristensen, Birger Brodin, Irena Loryan

**Affiliations:** 1 Department of Pharmacy, CNS Drug Delivery and Barrier Modelling group (CNSBM), University of Copenhagen, Copenhagen, Denmark; 2 Department of Pharmacy, Translational Pharmacokinetics/Pharmacodynamics group (tPKPD), Uppsala University, Uppsala, Sweden

**Keywords:** Migraine, Eletriptan, Sumatriptan, Blood-brain barrier, CNS, PNS, Central effect, 5-HT_1B/1D/1F_ receptors, Combinatory mapping approach, Free drug theory

## Abstract

**Background:**

Triptans are potent 5-HT_1B/1D/1F_ receptor agonists used in migraine therapy, thought to act through peripheral mechanisms. It remains unclear whether triptans cross the blood-brain barrier (BBB) sufficiently to stimulate central 5-HT_1B/1D/1F_ receptors. This study investigates the disposition of eletriptan and sumatriptan in central nervous system (CNS) and peripheral nervous system (PNS) regions and predicts regional 5-HT_1B/1D/1F_ receptor occupancies at clinically relevant concentrations.

**Methods:**

Using the Combinatory Mapping Approach (CMA) for regions of interest (ROI), we assessed the unbound tissue-to-plasma concentration ratio (K_p, uu, ROI_) in rats at steady state across CNS (hypothalamus, brain stem, cerebellum, frontal cortex, parietal cortex, striatum, hippocampus, whole brain, and spinal cord) and PNS (trigeminal ganglion and sciatic nerve) regions. We used K_p, uu, ROI_ values to estimate unbound target-site concentrations and 5-HT_1B/1D/1F_ receptor occupancies in humans.

**Results:**

We observed heterogenous triptan transport across CNS and PNS regions with the highest extent of unbound drug transport across the blood-nerve barrier in the trigeminal ganglion (K_p, uu, TG_: eletriptan: 0.519, and sumatriptan: 0.923). Both drugs displayed restricted entry across the BBB (K_p, uu, whole brain_: eletriptan: 0.058, and sumatriptan: 0.045) combined with high inter-regional variability. We estimated near-complete receptor occupancy in the trigeminal ganglion, while lower occupancies were observed in the whole brain, irrespective of the drug or receptor subtype. For instance, eletriptan was predicted to achieve 84% 5-HT_1B_ receptor occupancy in the trigeminal ganglion and 37% in the whole brain at clinically relevant concentrations.

**Conclusions:**

This study suggests that despite low BBB transport, both eletriptan and sumatriptan achieve unbound concentrations sufficient to stimulate 5-HT_1B,_ 5-HT_1D_, and 5-HT_1F_ receptors not only in the trigeminal ganglion, but also in the CNS. Further research is needed to determine whether central mechanisms contribute to triptan’s antimigraine effect and/or side effects.

**Graphical Abstract:**

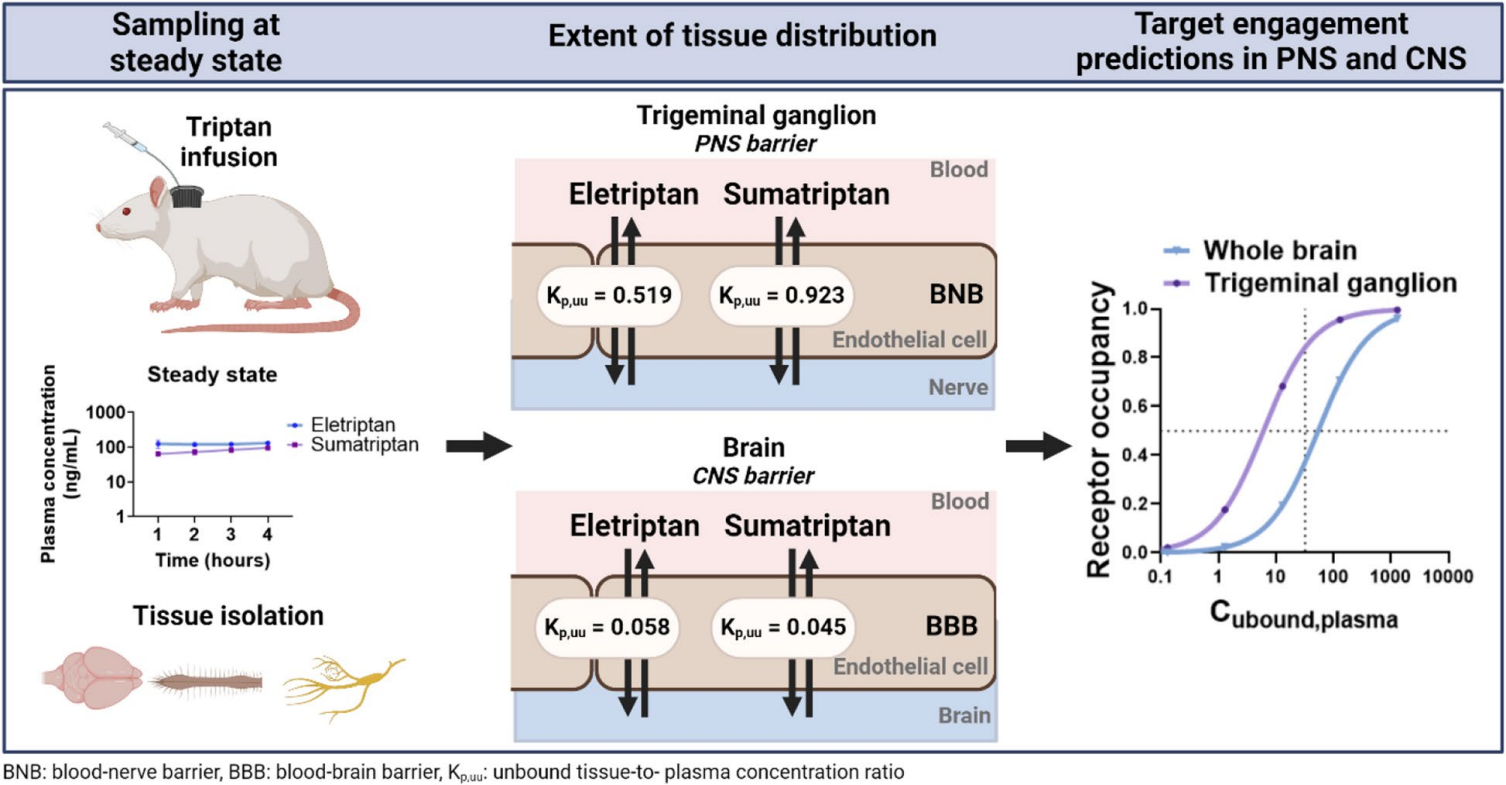

**Supplementary Information:**

The online version contains supplementary material available at 10.1186/s10194-024-01894-0.

## Background

Migraine is a common neurovascular disease affecting approximately 15% of the global population [1, 2]. Triptans are generally considered the most effective class of compounds for the treatment of acute migraine [3]. There are seven triptans in clinical use including sumatriptan, rizatriptan, eletriptan, zolmitriptan, almotriptan, frovatriptan, and naratriptan [4, 5]. Triptans act as potent agonists of the 5-hydroxytryptamine (5-HT) receptor subtype 1B, 1D, and 1F [6]. However, their exact mechanism and site of action remain a subject of ongoing debate [7–9].

**Fig. 1:**
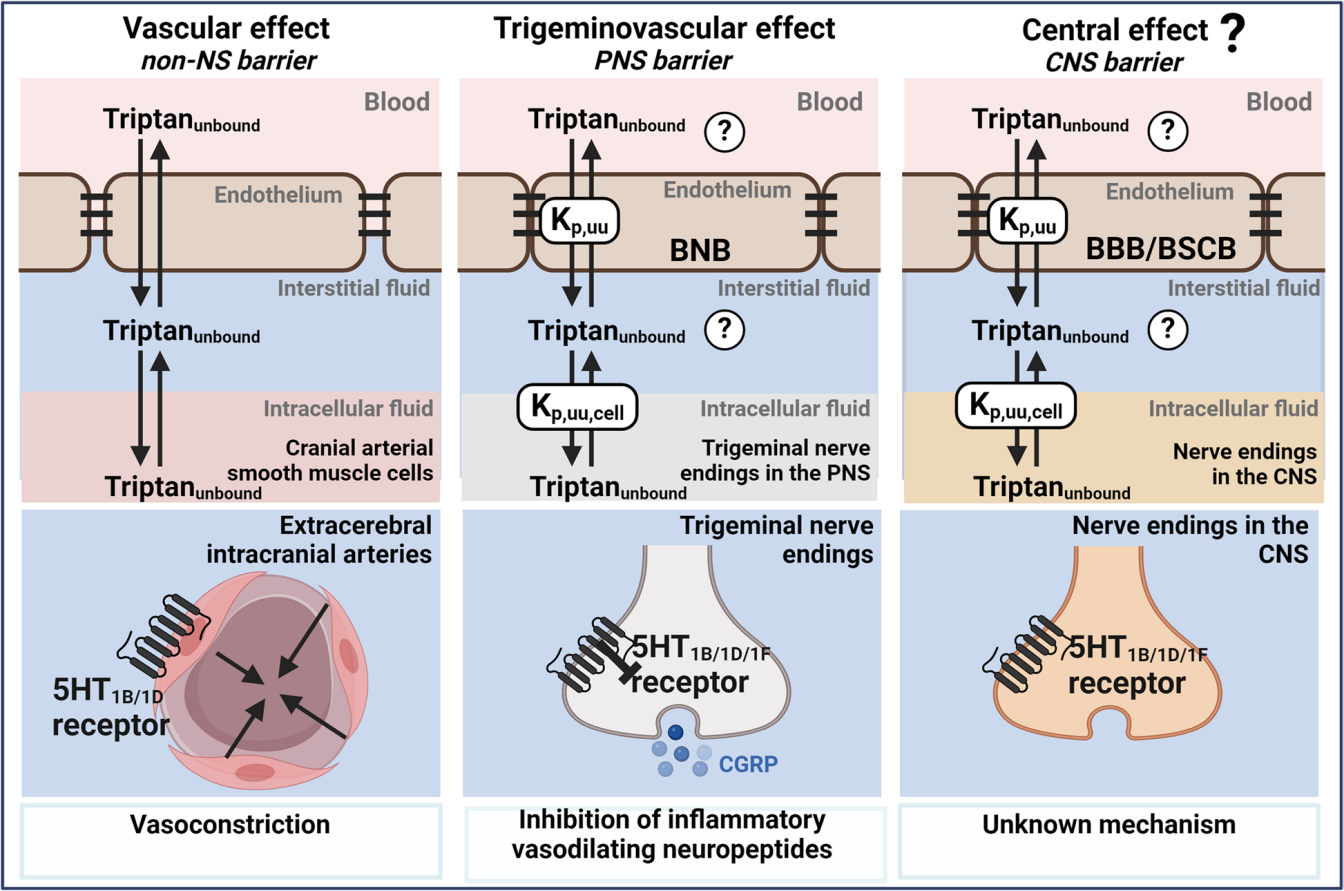
Schematic illustration of triptan pharmacology from a neuropharmacokinetic point of view. Unbound target-site concentrations are governed by interrelated and interconnected processes including the passage of unbound drug across the blood-to-tissue endothelial interfaces and cellular barriers. Triptans are known to exert their action through 5-HT_1B/1D/1F_ receptors. Their proposed mode of action involves vascular, trigeminovascular, and central mechanisms. For triptans to cause these actions, they need to cross endothelial barriers including the non-nervous system (NS) barriers, PNS barriers (BNB; blood-nerve barrier), and CNS barriers (BBB; blood-brain barrier or BSCB; blood-spinal cord barrier). K_p, uu_ is the unbound tissue-to-plasma concentration ratio describing the extent of unbound drug transport across an endothelial barrier and K_p, uu, cell_ is the unbound intracellular-to-extracellular (interstitial) concentration ratio describing the extent of cellular barrier transport. NB: for simplicity, only key pharmacodynamic mechanisms are illustrated

Originally, triptans were developed as vasoconstrictors targeting intracranial extracerebral arteries [9, 10]. Nowadays multiple mechanisms have been proposed to account for the antimigraine effect including vascular, trigeminovascular, and central mechanisms, which might act additively (Fig. 1) [10]. The predominant mechanism of action appears to be through trigeminal projections, which is situated in the peripheral nervous system (PNS) [6, 9–11]. Nonetheless, the presence of 5-HT_1B_, 5-HT_1D_, and 5-HT_1F_ receptors in various regions of the central nervous system (CNS) highlights that the CNS might also be a potential target area for triptans [12–16].

The PNS is protected by the peripheral blood-nerve barrier (BNB), while the brain is protected by the blood-brain barrier (BBB). The BBB is a highly restrictive barrier that separates the brain parenchyma from the circulatory system as reviewed by Abbott et al. 2010 [17]. In contrast, the BNB is considered less restrictive, as evidenced by higher paracellular transport of 4 kDa dextran and higher extent of the transport of small molecular weight drugs [18].

Triptans are relatively hydrophilic and positively charged at physiological pH. Given their hydrophilic nature and several indications of efflux transporter interactions, triptans are expected to have poor BBB penetrating properties [19–22]. Despite this, there is evidence to suggest that triptans reach the CNS to some extent. For instance, a recent systematic review highlights that triptans may cross the BBB based on diverse neuroimaging studies [23]. Muzzi et al. (2020) further demonstrated rapid brain uptake of sumatriptan in rats, with accumulation in the hypothalamus and brain stem within 1 and 5 min following subcutaneous injections [24]. Additionally, a meta-analysis study of oral triptans have reported CNS-related adverse events such as fatigue, somnolence, and dizziness, which imply CNS involvement [25, 26]. Whether triptans cross the BBB to an extent that significantly contributes to their pharmacological action remains an open question [8, 9, 23, 27, 28].

This study aims to address this question by examining two triptans: the more lipophilic eletriptan and the prototypical more hydrophilic sumatriptan. Specifically, we aim to assess the extent of eletriptan and sumatriptan transport into CNS regions (hypothalamus, brain stem, cerebellum, frontal cortex, parietal cortex, striatum, hippocampus, whole brain, spinal cord) and PNS regions (trigeminal ganglion and sciatic nerve). Additionally, we aim to predict the regional 5-HT_1B_, 5-HT_1D_, and 5-HT_1F_ receptor occupancy at clinically relevant triptan concentrations.

**Fig. 2:**
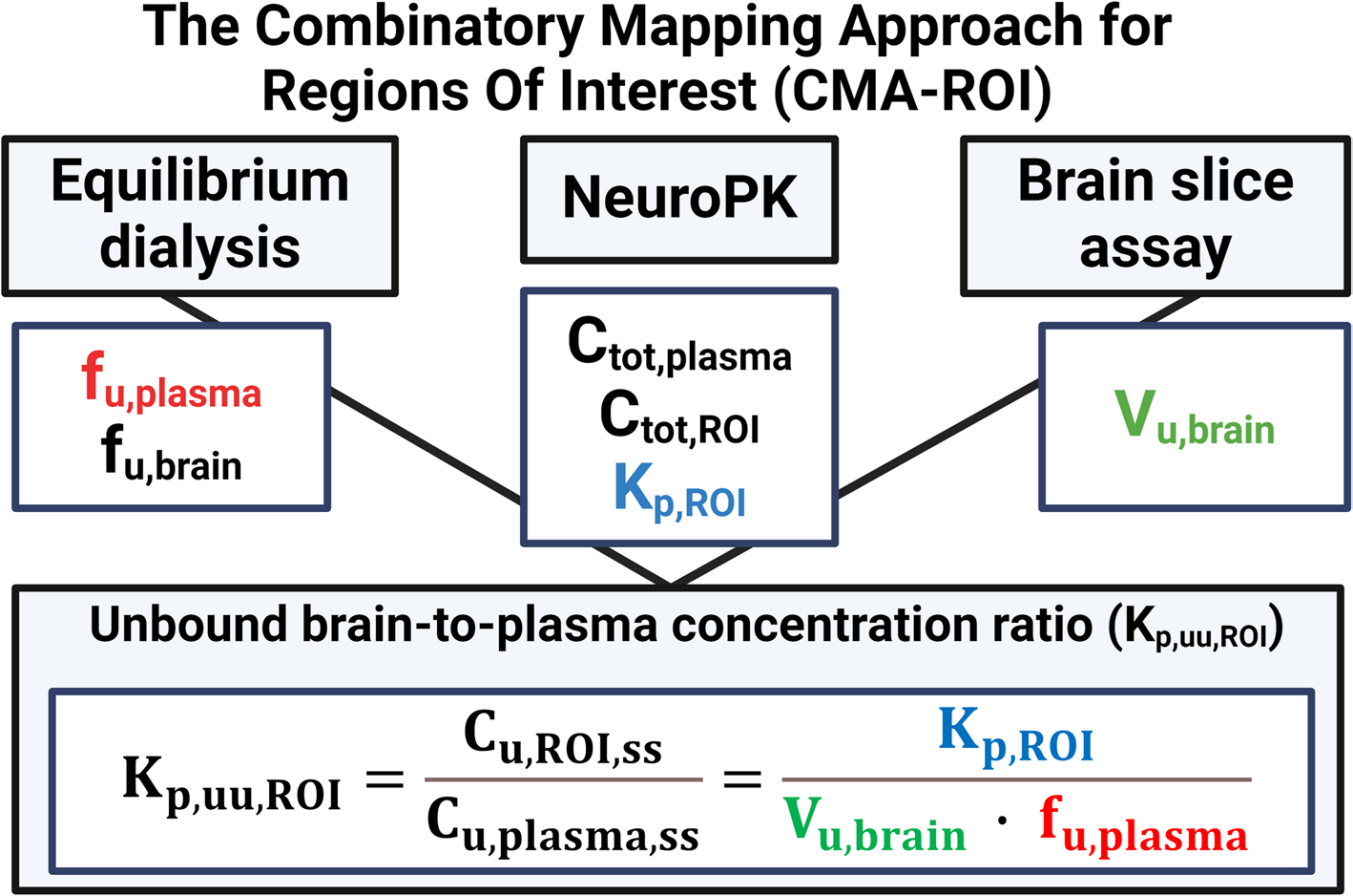
Schematic illustration of the CMA-ROI. The CMA-ROI combines preclinical neuropharmacokinetic parameters such as C_tot, plasma_, C_tot, ROI_, and K_p_, with in vitro equilibrium dialysis and brain slice techniques to determine drug binding properties in respective matrices (f_u,plasma_, f_u,brain_, f_u,nerve_) and unbound volume of distribution in the brain (V_u, brain_). Created with Biorender.com

Our approach is based on the free-drug theory, which proclaims that only unbound and unionized drug can pass membranes and engage with pharmacological targets [29]. We applied the Combinatory Mapping Approach for Regions of Interest (CMA-ROI), which integrates preclinical neuropharmacokinetic (neuroPK) parameters with information on drug binding in respective matrices measured in vitro using brain slice and equilibrium dialysis assays (Fig. 2) [30, 31]. The CMA-ROI allows the assessment of unbound target-site concentrations for calculations of the unbound tissue-to-plasma concentration ratio (K_p, uu_) [30, 31]. In addition, this approach allows the determination of the unbound intracellular-to-brain interstitial concentration ratio (K_p, uu, cell_) for the characterization of the extent of cellular barrier transport [32, 33].

Here, we determined unbound tissue and plasma concentrations in rats at steady state in order to calculate the K_p,uu_ in various PNS and CNS regions. The highest drug exposure was observed in the trigeminal ganglion revealing K_p, uu_ values of 0.519 for eletriptan and 0.923 for sumatriptan. These findings suggest that sumatriptan penetrates the BNB more readily, potentially through passive diffusion or mutually compensated efflux and influx, while eletriptan is subjected to moderate efflux. Both drugs entered the brain parenchyma with K_p, uu_ values of 0.058 for eletriptan and 0.045 for sumatriptan, suggesting a low extent of BBB transport due to significant active efflux. Notably, variability in K_p,uu_ was observed across different brain regions. Despite low BBB transport, our predictions suggest that both eletriptan and sumatriptan achieve unbound concentrations sufficient to stimulate 5-HT_1B/1D/1F_ receptors, not only in the trigeminal ganglion, but also in the CNS.

## Methods

### Chemicals

Sumatriptan succinate (99.94%), sumatriptan-d6 succinate (90%) and eletriptan hydrobromide (98.13%) were ordered from MedChemExpress (New Jersey, USA). Acetonitrile, gradient grade for liquid chromatography, and anhydrous dipotassium hydrogen phosphate were purchased from Merck (Darmstadt, Germany). Formic acid, reagent grade ≥ 95%, sodium chloride (NaCl), potassium chloride (KCl), magnesium sulfate (MgSO_4_), calcium dichloride (CaCl_2_), HEPES (N’-2-Hydroxyethylpiperazine-N’-2 ethane sulphonic acid), ascorbic acid, and potassium dihydrogen phosphate (KH_2_PO_4_) were purchased from Sigma-Aldrich (Steinheim, Germany), and dimethyl sulfoxide (DMSO) was purchased from MP Biomedicals (Eschwege, Germany). The Milli-Q Academic system (Millipore, Bedford, MA, USA; Resistance 18.2 Ohm; Millipak^®^Express 20 Filter, 0.22 μm) was purchased from Merck Millipore (Burlington, MA, USA).

### Animals

Healthy male and female Sprague-Dawley rats weighing 250–325 g (Taconic, Lille Skensved, Denmark) were used for all animal experiments. The selection of male and female rats was based on the intention to have a heterogeneous group of animals. The rats were individually housed by sex, provided with ad libitum access to food and water, and maintained under a 12-hour light-dark cycle at temperatures ranging from 20 to 22 °C and 45–65% humidity. All experiments adhered to the guidelines of the Swedish National Board for Laboratory Animals and were approved by the Animal Ethics Committee of Uppsala, Sweden (Ethical approval number: 5.8.18–12230/2019). The studies were non-blinded and non-randomized. The animal studies have been reported in agreement with the ARRIVE guidelines [34]. A minimally required sample size of four to six animals per group was calculated in order to achieve the desired statistical power for a two-tailed t-test study, given the *p*-value of 0.05, the anticipated effect size, i.e., Cohen’s d in the range of 2 to 2.5, and a desired statistical power level of 0.8.

### The Combinatory Mapping Approach for regions of interest (CMA-ROI)

The CMA combines in vivo and in vitro experiments to determine the unbound tissue-to-plasma concentration ratio (K_p, uu_) [30, 31]. The K_p, uu_ value is the state-of-the-art neuropharmacokinetic parameter describing the extent of drug transport across a barrier [35, 36]. This approach combines: (1) In vivo neuropharmacokinetic studies in rats to determine the plasma and tissue concentrations at steady state to determine the total tissue-to-plasma concentration ratio (K_p_), (2) in vitro equilibrium dialysis studies to determine unbound fractions in brain and plasma (f_u, brain_ and f_u, plasma_), and (3) in vitro brain slice assays to determine the unbound volume of distribution (V_u, brain_). In addition, the unbound intracellular-to-extracellular (interstitial) concentration ratio (K_p, uu, cell_) across brain parenchymal cells can be derived from the CMA. A schematic illustration of the CMA, its methodologies and output parameters are shown in Fig. 2.

### In vivo neuropharmacokinetic (neuroPK) study

In vivo neuroPK evaluation of eletriptan and sumatriptan was assessed in rats at steady state with the overall purpose of determining steady state tissue-to-plasma concentration ratios, K_p, brain_(Eq. 1). Male (*N* = 6) and female (*N* = 6) Sprague-Dawley rats were used for neuroPK experiments. Surgical implantation of catheters in the femoral vein and femoral artery for intravenous (IV) drug infusion and arterial blood sampling was done one day prior to the experiments. During the procedure, the animals were placed on a heating pad under anesthesia induced by 5% isoflurane and maintained by 2.5% isoflurane, supplemented with 3 L/min oxygen. After catheterization, the rats were individually transferred to a CMA120 system (CMA, Solna, Sweden). On the day of the experiment, awake rats were weighed, and the loading and maintenance doses were calculated based on individual weights (Table 1). The rats were initially given a 10-minute fast-rate IV loading dose followed by a constant 4-hour infusion. The dosing regimen was chosen based on publicly available pharmacokinetic parameters in rats (volume of distribution and clearance) to achieve a steady state plasma concentration corresponding to clinically relevant concentrations of 150–200 ng/mL [37, 38].

**Table 1:** A summary of used steady state intravenous infusion regimen for in vivo neuropharmacokinetic studies

Drug	Loading dose (µg/kg/min)	Maintenance dose (µg/kg/min)	Total dose (mg/kg)
Eletriptan	218	22	7.5
Sumatriptan	49	6.8	2.1

Blood samples were collected from the vein catheter before (0 h) and at 1, 2, 3, and 4 h after the start of the drug infusion and terminally by heart puncture. Plasma was isolated from the blood samples by centrifugation at 10,000 rpm for 5 min. After decapitation, the brain was isolated and dissected into two halves. One half was microdissected with isolation of regions of interest such as the frontal cortex, parietal cortex, cerebellum, hippocampus, hypothalamus, brain stem, and striatum. The second half was also collected and referred to as the whole brain. The spinal cord, sciatic nerve, and trigeminal ganglion were also sampled. The sciatic nerve was included as a comparable peripheral nerve tissue to the trigeminal ganglion due to its distinct anatomical location and ease of isolation. The dissected tissues were cleaned by removing large blood vessels. All samples were transferred to ceramic microbead-containing vials (VWR, Sweden), weighed, and immediately placed at -80 °C. Before analysis, tissue samples were mixed with 1:4 (w: v) phosphate-buffered saline pH 7.4 (PBS) and homogenized mechanically on a Mini Bead Mill (VWR, Sweden). Samples were immediately frozen and stored at -80 °C.

The K_p, ROI_ was calculated as:

1 $${\mathrm K}_{\mathrm p,\mathrm{ROI}}=\frac{{\mathrm C}_{\mathrm{tot},\mathrm{ROI},\mathrm{ss}}}{{\mathrm C}_{\mathrm{tot},\mathrm{plasma},\mathrm{ss}}}$$

Where C_tot,ROI,ss_ is the total concentration of a drug in tissue at steady state corrected for a residual drug in blood according to Fridén et al. [39], C_tot,plasma,ss_ is the total concentration of a drug in plasma at steady state.

Small volumes of contaminating blood may contribute to the overestimation of drug concentrations in tissue, particularly when investigating low BBB permeable drugs, where blood concentrations are significantly higher than brain concentrations. Hence, the total drug concentration in tissue corrected for residual blood was calculated as:

2 $${\mathrm C}_{\mathrm{tot},\mathrm{ROI},\mathrm{ss}}=\frac{{\mathrm C}_{\mathrm{tot},\mathrm{ROI},\mathrm{ss},\mathrm{uncorrected}}-{\mathrm V}_{\mathrm{eff}}{{.}\mathrm C}_{\mathrm{tot},\mathrm{plasma},\mathrm{ss}}}{1-{\mathrm V}_{\mathrm{water}}}$$

Where V_water_ is the apparent brain vascular volume of plasma water (10.3 µL/g brain), and V_eff_ is the effective plasma volume. V_eff_ was calculated as [39]:

3 $${\mathrm V}_{\mathrm{eff}}={\mathrm f}_{\mathrm u,\mathrm{plasma}}\cdot{\mathrm V}_{\mathrm{water}}+\left(1-{\mathrm f}_{\mathrm u,\mathrm{plasma}}\right)\cdot{\mathrm V}_{\mathrm{protein}}$$

Where f_u, plasma_ is the fraction of unbound drug in plasma, V_protein_ is the apparent brain vascular volume of plasma protein (7.99 µL/g brain) [39].

### Equilibrium dialysis

Assessment of the unbound fraction of eletriptan and sumatriptan in plasma (f_u,plasma_), brain (f_u,brain_), and sciatic nerve (f_u,nerve_) were performed by equilibrium dialysis using an HTD 96b equilibrium dialysis device (HTDialysis, CT, USA) [40]. This method takes advantage of the ability of unbound drug being able to penetrate a semipermeable membrane, while protein-bound drug cannot. The semipermeable membrane separates a protein-containing compartment from a protein-free buffer compartment allowing estimation of the extent of drug tissue binding properties at equilibrium. A schematic illustration of the equilibrium dialysis principle is shown in Fig. 4A. Equilibrium dialysis experiments were performed using three biological and three technical replicates unless otherwise stated. The two chambers of the equilibrium dialysis wells were separated by a semipermeable cellulose membrane with a cutoff of 12–14 kDa. The cellulose membrane was prepared according to the manufacturer’s recommendations (HTDialysis, CT, USA). Tissues were homogenized mechanically using microbead-containing vials on a Mini Bead Mill (VWR, Sweden) followed by further short homogenization using a Vibra Cell ultrasonic processor VCX-130 (Chemical instruments A/B, Sweden). The final concentration of eletriptan and sumatriptan was 1 µM in tissue homogenate, and 200 or 400 nM in plasma. The rationale for selecting a concentration of 1 µM in tissue homogenate was to ensure the quantification of highly bound drugs. Two concentrations were evaluated in plasma, matching expected plasma concentrations in neuroPK studies which were relatively compatible with clinically relevant human peak plasma concentrations [37, 38].

One chamber was filled with 100 µL of PBS. The second chamber was filled with 100 µL of a drug containing undiluted plasma or tissue homogenate 1:9 (w: v) brain or sciatic nerve homogenate in PBS. The dialysis was running for 6 h on orbital shaker at 37 °C, 200 rpm. After 6 h, 50 µL samples were taken from the buffer and plasma/tissue compartments. Buffer samples were added to an equal amount of plasma/tissue, while plasma/tissue samples were added to an equal amount of buffer to match the matrices required for bioanalysis. Samples were immediately frozen and stored at -80 °C.

Thermostability and recovery of the compounds were controlled in plasma or brain homogenate in parallel with the experiment. Samples were taken before and after the 6-hour incubation at 37 °C, 200 rpm. A thermostability of 100 ± 30% of the initial drug concentration was accepted. The thermostability was calculated as:

4 $$\mathrm{Termostability}\;\left(\%\right)=\frac{{\mathrm C}_{6\;\mathrm{hours}}}{{\mathrm C}_{0\;\mathrm{hours}}}\cdot100\%$$

Where C_0 hours_ and C_6 hours_ are the concentrations in plasma/brain homogenate before and after incubation.

The recovery of the spiked drug concentration into the plasma/brain homogenate was calculated in percentage of theoretical drug concentrations to evaluate potential sticking to the plastic. The recovery was calculated as:

5 $$\mathrm{Recovery}\;\left(\%\right)\frac{{\mathrm C}_{0\;\mathrm{hours}}}{{\mathrm C}_{\mathrm{theoretical}}}\cdot100\%$$

Where C_theoretical_ is the actual spiked buffer concentration.

The unbound fraction of drug in plasma (f_u,plasma_) was calculated as:

6 $${\mathrm f}_{\mathrm u,\mathrm{plasma}}=\frac{{\mathrm C}_{\mathrm{buffer}}}{{\mathrm C}_{\mathrm{plasma}}}$$

Where C_buffer_ represents the drug concentration in the buffer/receiver compartment and C_plasma_ represents the drug concentration in the plasma/donor compartment at the end of 6-hour incubation.

The unbound fraction of drug in diluted brain homogenate (f_u,D,brain_) was calculated as:

7 $${\mathrm f}_{\mathrm u,\mathrm D,\mathrm{brain}}=\frac{{\mathrm C}_{\mathrm{buffer}}}{{\mathrm C}_{\mathrm{brain}}}$$

Where C_brain_ represents the drug concentration in the diluted brain homogenate/donor compartment.

To account for the dilution of brain homogenate, the unbound fraction of drug in undiluted brain homogenate (f_u, brain_) was calculated as:

8 $${\mathrm f}_{\mathrm u,\mathrm{brain}}=\frac{\displaystyle\frac1{\mathrm D}}{\left({\displaystyle\frac1{{\mathrm f}_{\mathrm u,\mathrm D,\mathrm{brain}}}}-1\right)+{\displaystyle\frac1{\mathrm D}}}$$

Where D represents the dilution factor of the homogenate in PBS and is equal to 10 in this experiment.

### In vitro brain slice assay

Assessment of the intracellular distribution of unbound eletriptan and sumatriptan was performed by the in vitro brain slice method [41, 42]. A schematic illustration of the brain slice principle is shown in Fig. 4D. Artificial extracerebral fluid (aECF) was prepared using ultra-pure water (Millipore, MA, USA) supplemented with 129 mM NaCl, 3 mM KCl, 1.2 mM MgSO_4_, 25 mM HEPES, K_2_HPO_4_, 1.4 mM CaCl_2_, 0.4 mM ascorbic acid, and 10 mM glucose, and adjusted to pH 7.3 at 37 °C. The aECF was equilibrated with 100% oxygen for 15 min. The brain was isolated from naïve male rats (*N* = 3 per drug). Coronal slices of the brain were performed using a vibrating blade microtome (Leica Microsystems AB, Sweden) with a slice thickness of 300 μm. Six slices were transferred to a beaker with ice-cold aECF for approximately 5 min. The brain slices were transferred to a beaker with 15 mL of prewarmed aECF containing 100 nM eletriptan or sumatriptan and sealed with a breathable film (Diversified Biotech MA, USA). The brain slices in aECF were transferred to a benchtop orbital shaker (MaxQ4450, Thermo Scientific, MA, USA) and incubated for 5 h at 37 °C, 45 rpm, and 75–80 mL O_2_/min. The pH of the aECF containing brain slices was between 7.2 and 7.4 after 5-hour incubation. The aECF buffer was sampled from the brain slice containing beaker after the achievement of equilibrium at 5 h (C_buffer_). All aECF samples were mixed 1:1 (v: v) with 1:4 (w: v) brain homogenate in aECF. Brain slices were dried on filter paper, individually weighed, and homogenized with 1:9 (w: v) of aECF using a Vibra Cell ultrasonic processor VCX-130 (Chemical instruments A/B, Sweden) for 5 s at 50% amplitude. Samples were immediately frozen and stored at -20 °C.

The thermostability of the compounds was assessed in parallel with the experiment. Samples were taken from a solution without brain slices before and after the 5-hour incubation at 37 °C, 45 rpm. A thermostability of 100 ± 30% of the initial drug concentration was accepted.

9 $$\mathrm{Termostability}\;\left(\%\right)=\frac{{\mathrm C}_{5\;\mathrm{hours}}}{{\mathrm C}_{0\;\mathrm{hours}}}\cdot100\%$$

Where C_0 hours_ and C_5 hours_ are the concentrations in buffers before and after incubation. The recovery of the spiked drug concentration into the buffer solution was calculated according to Eq. 5.

Assuming that the drug concentration in the protein free aECF corresponds to the concentration in the brain interstitial fluid (ISF) at equilibrium, the V_u, brain_ was calculated as:

10 $${\mathrm V}_{\mathrm u,\mathrm{brain}}=\frac{{\mathrm A}_{\mathrm{brain}}}{{\mathrm C}_{\mathrm u,\mathrm{brain}\;\mathrm{ISF}}}\approx\frac{{\mathrm A}_{\mathrm{brain}}}{{\mathrm C}_{\mathrm{buffer}}}=>\frac{{\mathrm A}_{\mathrm{brain}}-{\mathrm V}_{\mathrm i}\cdot{\mathrm C}_{\mathrm{buffer}}}{{\mathrm C}_{\mathrm{buffer}}\cdot\left(1-{\mathrm V}_{\mathrm i}\right)}$$

Where A_brain_ represents the drug amount per g brain in the brain slice, C_buffer_ represents the dug concentration in aECF, V_i_ represents the leftover volume of the buffer on the brain slice and has previously been determined to 0.133 mL/g brain [18].

### NeuroPK parameters

Determined parameters from in vivo and in vitro studies were applied to determine key neuroPK parameters. The unbound tissue-to-plasma concentration ratio (K_p, uu_) was calculated as:

11 $${\mathrm K}_{\mathrm p,\mathrm{uu},\mathrm{tissue}}=\frac{{\mathrm K}_{\mathrm p,\mathrm{tissue}}}{{\mathrm V}_{\mathrm u,\mathrm{brain}}\cdot{\mathrm f}_{\mathrm u,\mathrm{plasma}}}$$

The observed unbound intracellular-to-interstitial concentration ratio (K_p,uu,cell,obs_) can be estimated based on f_u,brain_ and V_u,brain_ [32]. A schematic illustration of the brain slice principle is shown in Fig. 4F The K_p,uu,cell_ was calculated as:

12 $${\mathrm K}_{\mathrm p,\mathrm{uu},\mathrm{cell},\mathrm{obs}}=\frac{{\mathrm C}_{\mathrm u,\mathrm{ICF},\mathrm{ss}}}{{\mathrm C}_{\mathrm u,\mathrm{ISF},\mathrm{ss}}}={\mathrm V}_{\mathrm u,\mathrm{brain}}\cdot{\mathrm f}_{\mathrm u,\mathrm{brain}}$$

Where C_u,ICF_ is the unbound concentration in the intracellular fluid (ICF) and C_u,ISF_ is the unbound concentration in the ISF [34].

The unbound cell partitioning ratio can be predicted (K_p,uu,cell,pred_) based on the assumption that only unbound and unionized drug can pass through the cellular membrane by passive diffusion mechanism [33]. Using physiological volumes and pH of ISF, cytosol and lysosomes as well as predicted pK_a_ values of drugs, the K_p,uu,cell,pred_ can be predicted as:

13 $${\mathrm K}_{\mathrm p,\mathrm{uu},\mathrm{cell},\mathrm{pred}}={\mathrm V}_{\mathrm{ISF}}+{\mathrm K}_{\mathrm p,\mathrm{uu},\mathrm{cyto},\mathrm{pred}}\cdot\left({\mathrm V}_{\mathrm{cyto}}+{\mathrm V}_{\mathrm{lyso}}\cdot{\mathrm K}_{\mathrm p,\mathrm{uu},\mathrm{lyso},\mathrm{pred}}\right)$$

Where V_ISF_ is 0.2 mL/g brain, V_cyto_ is 0.79 mL/g brain, and V_lyso_ is 0.01 mL/g brain [33].

The K_p, uu, cytosol, pred_ can be calculated as:

14 $${\mathrm K}_{\mathrm p,\mathrm{uu},\mathrm{cyto},\mathrm{pred}}=\frac{10^{\left(\mathrm{pKa}-{\mathrm{pH}}_{\mathrm{cyto}}\right)}+1}{10^{\left(\mathrm{pka}-{\mathrm{pH}}_{\mathrm{ISF}}\right)}+1}$$

Where pH_cytosol_ is 7.02, pH_ISF_ is 7.3 [33], predicted pKa_eletriptan_ is 8.4, and predicted pKa_sumatriptan_ is 9.5 [43].

The K_p, uu, lyso, pred_ of basic drugs can be calculated as:

15 $${\mathrm K}_{\mathrm p,\mathrm{uu},\mathrm{lyso},\mathrm{pred}}=\frac{10^{\left(\mathrm{pKa}-{\mathrm{pH}}_{\mathrm{lyso}}\right)}+1}{10^{\left(\mathrm{pka}-{\mathrm{pH}}_{\mathrm{cyto}}\right)}+1}$$

Where pH_lyso_ is 5.18 [33].

### Sample preparation and bioanalysis

Quantification of sumatriptan and eletriptan in plasma and tissues was conducted using ultra-performance liquid chromatography coupled with tandem mass spectrometry (UPLC-MS/MS). The method for the assessment of plasma and tissue concentrations was developed based on previously published protocols with modifications [22, 44–52]. Total sumatriptan and eletriptan in plasma and tissue samples were quantified using Acquity UPLC coupled with Xevo TQ-S Micro triple quadrupole mass spectrometer (MS/MS) (Waters Corporation, Milford, MA, USA). The bioanalytical method parameters and sample preparation details are described in Additional file 1. Acceptance criteria were predefined according to the FDA guidance [53]. Sumatriptan, eletriptan and internal standard sumatriptan-d6 were analyzed in positive electrospray ionization mode with multiple reaction monitoring (MRM) mode to monitor Parent → Product ion (m/z) transitions, respectively. Preparation of plasma and tissue samples, respective standards, quality control samples, and blanks, plasma, and tissue homogenates, were conducted in two steps. Plasma and tissue homogenate samples, with respective blanks, standards and quality controls were precipitated in acetonitrile 1:3 (v: v) with internal standard (sumatriptan-d6) followed by centrifugation. The supernatant was diluted 1:2 (v: v) in the mobile phase (MP) A, i.e., 0.1% formic acid in ultra-pure water.

The chromatographic separation of analytes was conducted using ACQUITY UPLC HSS C18 column, 2.1 × 100 mm, 1.8 μm, protected by an ACQUITY UPLC BEH C18 VanGuard pre-column, 2.1 × 5 mm, 1.7 μm (Waters Corporation, Milford, MA, USA) at 30 °C with a flow rate of 0.3 mL/min. MPA; 0.1% formic acid, and MPB; 0.1% formic acid in acetonitrile, were employed in gradient for separation of analytes and their elution. Details on mobile phase gradient can be found in Additional file 1.

The linear range with a determination coefficient equal to or higher than 0.99 was obtained in all standard curves prepared in the respective control matrices using the cassette approach, i.e., including sumatriptan and eletriptan at each standard level. The concentration range in the standard curves was 0.5–220 ng/mL for both sumatriptan and eletriptan (10 levels) in plasma and blank brain homogenate solution, and five levels of quality control samples for both matrices were prepared with concentrations of sumatriptan and eletriptan of 0.8 ng/mL, 4.0 ng/mL, 40 ng/mL, 80 ng/mL, 120 ng/mL, respectively. A 2-1000 nM (8 levels) range was employed for the equilibrium dialysis and brain slice assay experiments. The lowest standard points were set as limits of quantification. Data quantification was done using Masslynx v4.2 (Waters Corporation, Milford, MA, USA).

### Prediction of receptor occupancy based on neuropharmacokinetic data

Prediction of receptor occupancies (RO) was based on rat K_p,uu_ values and publicly available equilibrium dissociation constants (K_d_) and was assessed according to Hill’s equation:

16 $${\mathrm{RO}}_{\mathrm{ROI}}=\frac{{\mathrm C}_{\mathrm u,\mathrm{ROI}^{\mathrm n}}}{{\mathrm C}_{\mathrm u,\mathrm{ROI}^{\mathrm n}}+K_d}$$

Where C_u, ROI_ is the unbound concentration of a drug at ROI since only unbound drug is available for target engagement, *n* is Hill’s coefficient, which was set to 1.0, supported by reported values [54]. In the present study, i*n vitro* K_d_ and K_i_ values for human 5-HT_1B/1D_ and 5-HT_1F_ receptors, respectively were obtained from Napier et al. [55].

Unbound target-site concentrations (C_u,ROI_) in humans were as follows:

17 $${\mathrm C}_{\mathrm u,\mathrm{ROI}}={\mathrm C}_{\mathrm{tot},\mathrm{plasma}}\cdot{\mathrm f}_{\mathrm u,\mathrm{plasma}}\cdot{\mathrm K}_{\mathrm p,\mathrm{uu},\mathrm{ROI}}$$

Where C_tot,plasma_ is the plasma concentration in humans selected based on publicly available data, f_u, plasma_ is the fraction of unbound drug in plasma in humans (Table 2), and K_p,uu,ROI_ is unbound brain-to-plasma concentration ratio assessed in rats.

**Table 2:** Prediction of 5-HT_1B_, 5-HT_1D_, and 5-HT_1F _receptor occupancy levels in the PNS and CNS. The trigeminal ganglion (TG) represents the PNS region, while whole brain (WB) represents the CNS region. Predictions are based on unbound maximal target site concentrations after therapeutic doses of eletriptan and sumatriptan in humans

Parameter	Unit	Site	Eletriptan	Sumatriptan	
			PO 40 mg	PO 50 mg	PO 100 mg
K_d,5−HT1B_ [51]	nM	NA	3.14	11.07	
K_d,5−HT1D_ [51]	nM	NA	0.92	6.58	
K_i,5−HT1F_ [51]	nM	NA	10.23	13.18	
f_u, plasma_ [50]			0.13	0.66	
C_tot, max_ [35, 36]	nM or (*ng/mL)*	Plasma	246 (*94.0*)	102 (*30.1*)	180 (*53.2*)
C_u, max_	nM or (*ng/mL)*	Plasma	32.2 (*12.3*)	67.3 (*19.9*)	119 (*35.1*)
C_u, max, ROI_	nM	TG	16.6	62.1	109.8
C_u, max, ROI_	nM	WB	1.86	3.00	5.31
RO_5−HT1B_	%	TG	84	85	90
RO_5−HT1B_	%	WB	37	21	32
RO_5−HT1D_	%	TG	95	90	94
RO_5−HT1D_	%	WB	67	31	45
RO_5−HT1F_	%	TG	62	82	89
RO_5−HT1F_	%	WB	15	19	29

*PO* peroral, *TG* trigeminal ganglion, *WB* whole brain, *RO* receptor occupancy. The C_u,max,ROI_ has been calculated according to Eq. 17. The RO has been calculated according to Eq. 16. *NA* not applicable

### Statistical analysis

Statistical analysis and figure formatting were performed using GraphPad Prism 10.2.0 (CA, USA). Data are presented as mean ± standard deviation (SD). n denotes biological replicates; N denotes technical replicates. Normality test performance of each dataset was performed by a Shapiro-Wilk test. If stated, groups were compared using two-tailed unpaired student’s t-test or by a two-way ANOVA analysis followed by a Tukey’s multiple comparison test. *P*-values < 0.05 were considered statistically significant where *:*p* ≤ 0.05. **: *p* ≤ 0.01. ***: *p* ≤ 0.001. ****: *p* ≤ 0.0001.

## Results

### Heterogeneous transport across PNS and CNS barriers with the highest triptan exposure in the PNS

**Fig. 3:**
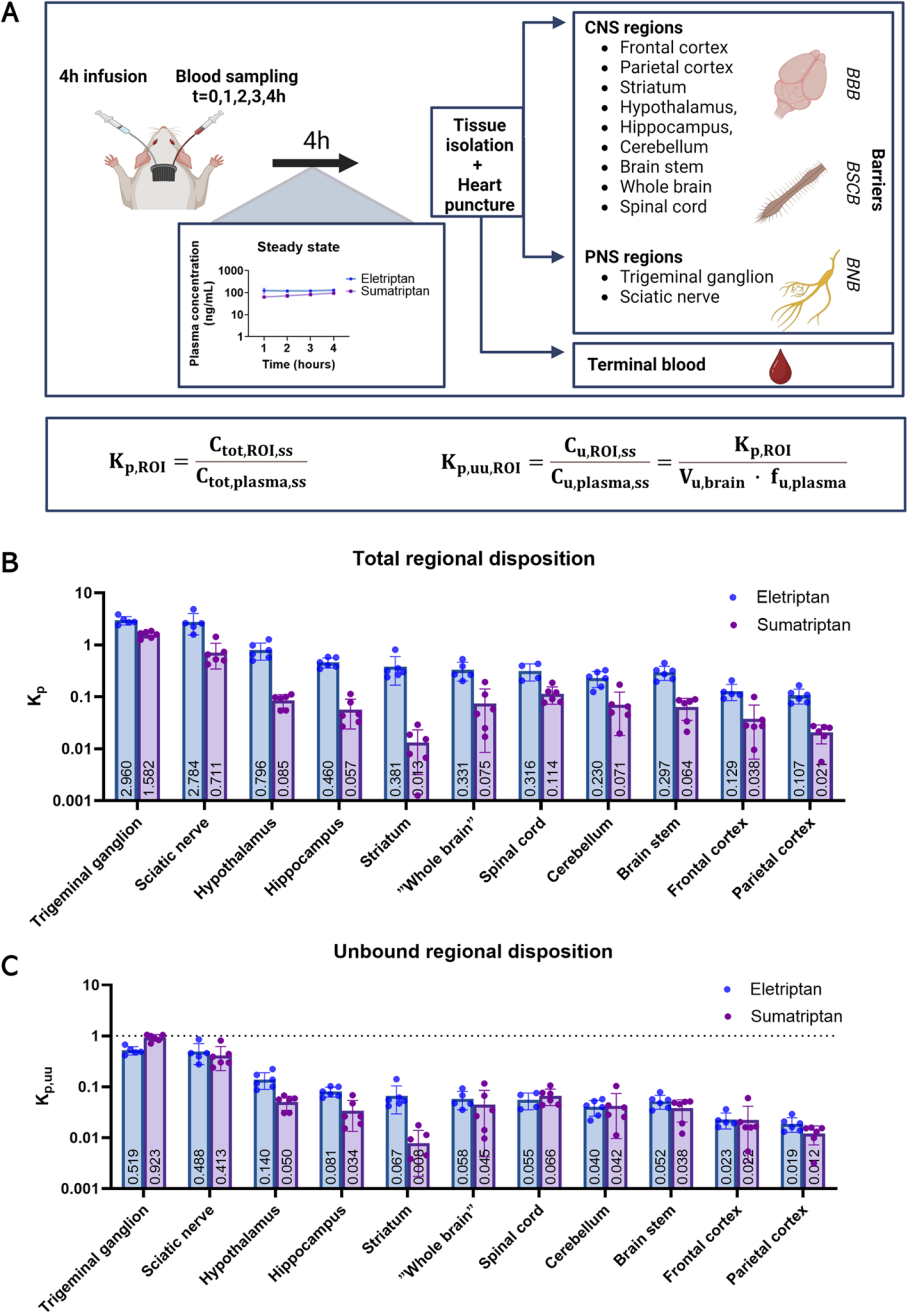
Assessment of regional K_p _and K_p, uu _for eletriptan and sumatriptan in rats under steady state.** A** Schematic overview of the neuropharmacokinetic study. Male and female rats were given a 4-hour IV infusion of eletriptan or sumatriptan to achieve steady state conditions. After 4 h, blood samples were collected terminally by heart puncture and tissues of interest were isolated including CNS regions: spinal cord, hypothalamus, striatum, cerebellum, brain stem, frontal cortex, and parietal cortex as well as whole brain, and PNS regions: trigeminal ganglion and sciatic nerve. Created with Biorender.com. **B** Total tissue-to-plasma concentration ratio (K_p_). **C** The unbound tissue-to-plasma concentration ratio (K_p, uu_). Columns represent mean ± SD (*n* = 4–6). The mean value of each column is annotated within each bar. The dotted line represents the line of unity. Values below unity indicate predominant active efflux across the respective barriers. In Fig. 3B and C, regions are sorted according to descending K_p_/K_p,uu_ values for eletriptan. NB: in B and C a semilogarithmic scale is used for the visualization of data. Data are presented with a linear scale in Additional File 3

We assessed the total drug concentrations of eletriptan and sumatriptan in CNS regions, PNS regions, and in plasma under steady state as shown in Fig. 3A. Eletriptan and sumatriptan achieved steady state plasma concentrations after a 4-hour dosing regimen (Additional file 2 A) reaching targeted mean C_tot, plasma_ of 123 ± 14 and 106 ± 39 ng/mL, respectively (Additional file 2B). The 4-hour infusion regimen differs from typical clinical practice. However, it was implemented to ensure achievement of steady state concentrations essential for the calculation of K_p_ and K_p, uu_.

Eletriptan demonstrated K_p, ROI_ values ranging from 0.107 to 2.960, with the highest values observed in the trigeminal ganglion followed by the sciatic nerve, hypothalamus, hippocampus, striatum, whole brain, spinal cord, cerebellum, brain stem, frontal cortex, and parietal cortex. Sumatriptan showed K_p, ROI_ values ranging from 0.021 to 1.582, with the highest values observed in the trigeminal ganglion followed by the sciatic nerve, spinal cord, hypothalamus, whole brain, cerebellum, brain stem, hippocampus, frontal cortex, parietal cortex, and striatum. Eletriptan exhibited higher K_p_ values than sumatriptan across all investigated regions (Fig. 3B). By exclusively considering the partitioning of unbound drug (K_p,uu_), we noted a similar distribution between the two triptans (Fig. 3C). Eletriptan demonstrated K_p,uu,ROI_ values ranging from 0.019 to 0.519, while sumatriptan demonstrated K_p, uu, ROI_ values ranging from 0.008 to 0.923. Both drugs showed limited entry into the brain ISF, with K_p,uu,whole brain_ values of 0.058 for eletriptan and 0.045 for sumatriptan. Among the brain regions, we found a 7.5-fold difference in K_p, uu_ for eletriptan ranging from the lowest in the parietal cortex to the highest in hypothalamus. For sumatriptan, we found a 6.4-fold inter-brain regional difference in K_p, uu_ ranging from the lowest in the striatum and the highest in hypothalamus.

Overall, these findings show that eletriptan and sumatriptan are profoundly influenced by the type of endothelial barrier, with increased drug exposure observed in tissues protected by the BNB compared to those protected by the BBB.

### Eletriptan and sumatriptan displayed differences in tissue binding and unbound volume of distribution in the brain but similar cellular partitioning

**Fig. 4:**
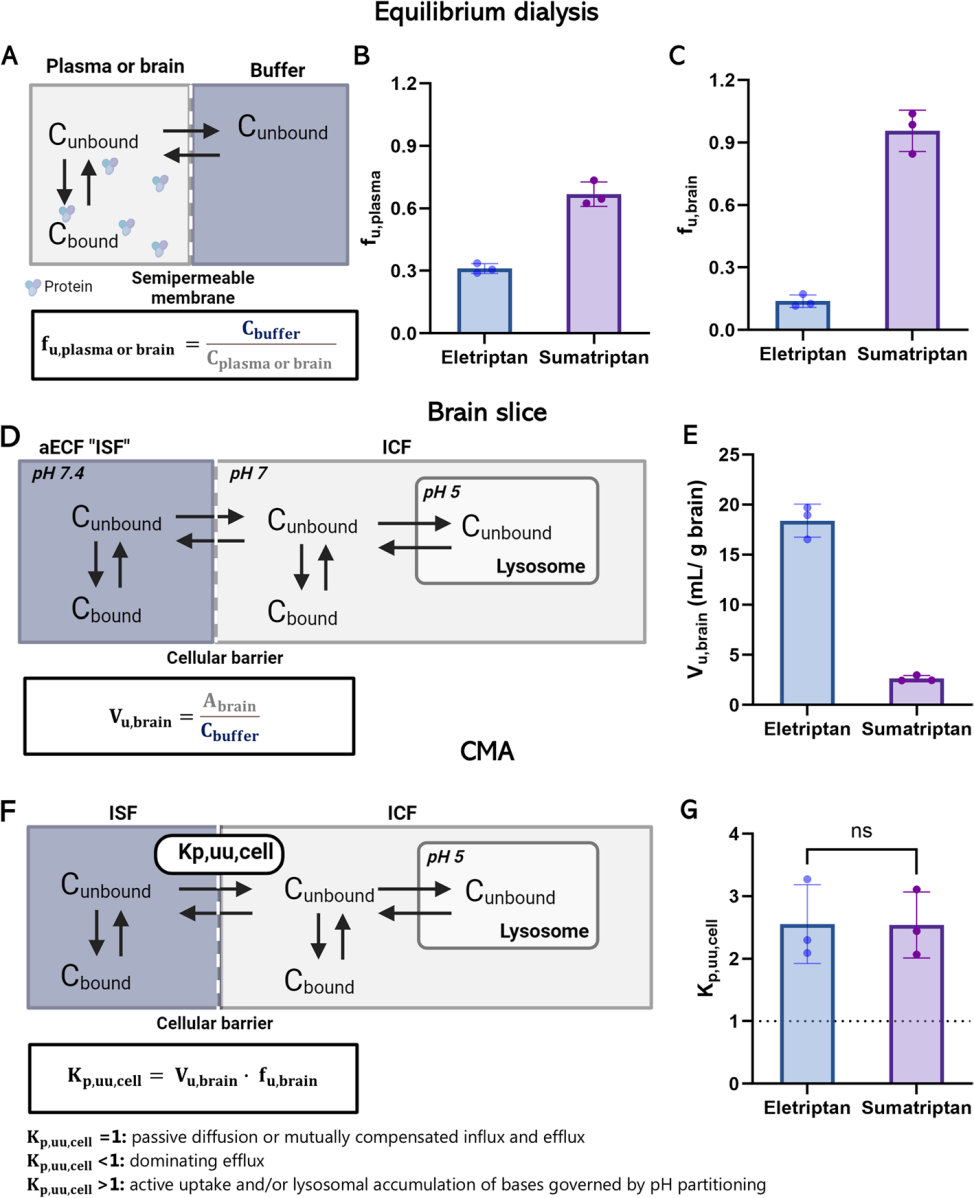
Plasma and brain tissue binding, unbound volume of distribution, and cellular partitioning in rat tissue.** A** Schematic illustration of the principle behind equilibrium dialysis. Only unbound drug (C_unbound_) can penetrate the semipermeable membrane allowing estimation of the extent of drug tissue binding at equilibrium. **B** The f_u,plasma_ of eletriptan and sumatriptan assessed at 200 nM (*n* = 3, *N* = 3). **C** The f_u, brain_ eletriptan and sumatriptan in brain homogenate assessed at 1 µM (*n* = 3, *N* = 3). **D** Schematic illustration of the principle behind V_u,brain_. The distribution of unbound drug from artificial extracellular fluid (aECF), which mimics brain ISF, into the ICF of brain parenchymal cells allows for transmembrane pH gradients, also enabling lysosomal trapping. **E** The V_u,brain_ of eletriptan and sumatriptan (100 nM) in rat brain slices (*n* = 3, *N* = 5). **F** Schematic illustration of the principle behind K_p, uu, cell_. **G **Estimated K_p, uu, cell_ of eletriptan and sumatriptan at equilibrium. In Fig. 4G, the dotted line represents the line of unity. Each column represents mean ± SD. The groups were compared using a student’s two-tailed t-test (*n* = 3, *N* = 5). Created with Biorender.com

Unbound fractions of eletriptan and sumatriptan in plasma (f_u,plasma_) and in brain homogenate (f_u,brain_) were determined using equilibrium dialysis. We found moderate plasma protein binding of eletriptan and sumatriptan as indicated by mean f_u,plasma_ of 0.31 and 0.67, respectively. These values were obtained using clinically relevant plasma concentrations of 200 nM, matching the concentrations achieved in the neuroPK studies (Fig. 4B). At a higher concentration of 400 nM, the f_u,plasma_ of eletriptan remained unchanged, while a 1.3-fold increase (*p* < 0.01) was found for sumatriptan (Additional File 4). In the brain, eletriptan revealed a f_u, brain_ of 0.14, while sumatriptan remained completely unbound with a f_u, brain_ approaching 1.0 (Fig. 4C). To ensure uniform non-specific binding across tissues, we assessed the binding properties of eletriptan and sumatriptan in nerve and brain tissue, finding no significant differences (Additional file 5). This supports the use of brain homogenate binding as a surrogate for nerve tissue binding for these compounds, consistent with previous studies [18, 56].

The V_u,brain_ was assessed using brain slice assays. This parameter accounts for both brain tissue binding and intracellular uptake allowing transmembrane pH gradients across intact cell membranes. A schematic illustration of the brain slice principle is shown in Fig. 4D. Eletriptan exhibited significantly higher distribution into brain tissue as compared to sumatriptan, with mean V_u,brain_ of 18.4 mL/g brain and 2.6 mL/g brain, respectively (Fig. 4E).

We estimated the intracellular-to-extracellular cell partitioning coefficients (K_p,uu,cell_), derived from f_u,brain_ and V_u,brain_ according to Eq. 12 [32, 33]. K_p,uu,cell_ describes the extent of transport across cellular membranes, reflecting the involvement of active transport processes (Fig. 4F). Despite significant differences in non-specific binding and unbound volume of distribution in the brain, eletriptan and sumatriptan showed similar K_p,uu,cell_ values of 2.6 and 2.5, respectively. A K_p,uu,cell_ higher than unity indicates intracellular accumulation governed by active uptake or lysosomal trapping in brain parenchymal cells (Fig. 4G). To evaluate whether the observed intracellular accumulation is a result of pH partitioning into the more acidic intracellular and lysosomal environment, we predicted the K_p,uu,cell,pred_ by assuming that only unbound and unionized drug can penetrate cellular membranes via passive diffusion according to Eq. 13 [33]. K_p,uu,cell,pred_ was found to be 2.9 for eletriptan and 3.0 for sumatriptan. The K_p,uu,cell, pred_ are in line with the experimental determined K_p,uu,cell_, suggesting that the intracellular accumulation is due to pH partitioning rather than active uptake.

Overall, these findings prove that eletriptan exhibits stronger non-specific binding across the investigated matrices compared to sumatriptan, but similar cell partitioning.

### Predicted 5-HT_1B/1D/1F_ receptor occupancies indicate CNS target engagement at clinically relevant triptan concentrations

**Fig. 5:**
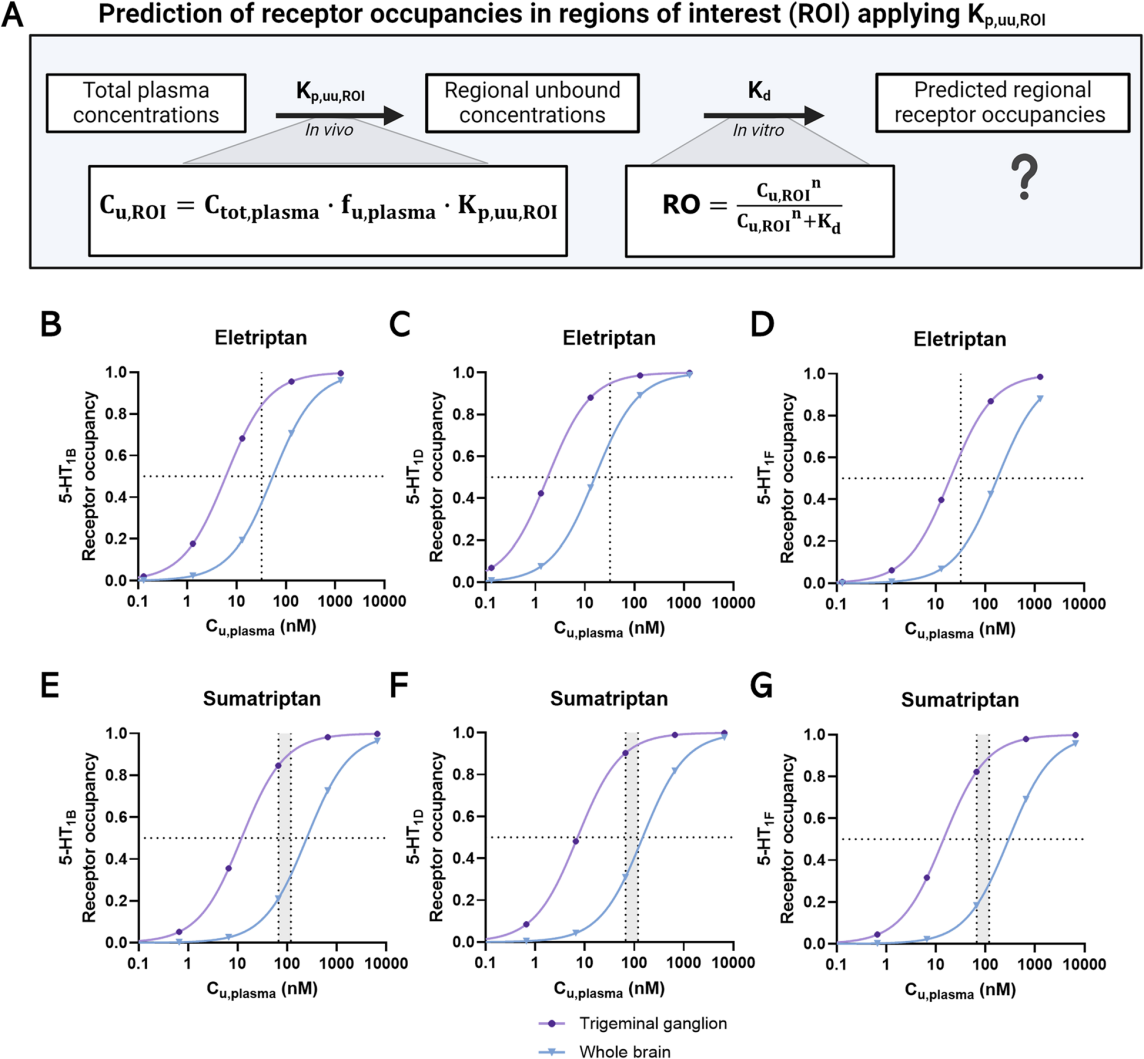
Predicted 5-HT_1B,_5-HT_1D_, and 5-HT_1F _receptor occupancies in the trigeminal ganglion and whole brain in humans. **A** Schematic illustration of the principle used to predict unbound target-site concentrations, applying K_p, uu, ROI_ and in vitro affinity constants to estimate potential target engagement. Created with Biorender.com. Binding dissociation constants, K_d_ (5-HT_1B_ and 5-HT_1D_) or K_i_ (5-HT_1F_) (human), were obtained from Napier et al. (1999) [55]. Receptor occupancies were predicted according to Eq. 16 and plotted against a range of unbound plasma concentrations to obtain full concentration-receptor occupancy profiles [54]. The horizontal dotted lines represent the 50% fractional receptor occupancy levels. The vertical dotted lines represent reported mean therapeutic C_max_ as unbound concentrations of eletriptan and sumatriptan after 40 mg PO or 50 mg/100 mg PO, respectively, in humans [37, 38]. **B** The predicted 5-HT_1B_ receptor occupancy of eletriptan in humans. **C** The predicted 5-HT_1D_ receptor occupancy of eletriptan in humans. **D** The predicted 5-HT_1F_ receptor occupancy of eletriptan in humans **(E)** The predicted 5-HT_1B_ receptor occupancy of sumatriptan in humans. **F** The predicted 5-HT_1D_ receptor occupancy of sumatriptan in humans. **G** The predicted 5-HT_1F_ receptor occupancy of sumatriptan in humans. All predicted receptor occupancies have been fitted to non-linear regression [agonist] vs. response with variable slope (four parameters)

Prediction of unbound target-site concentrations with linkage to in vitro potencies, is a well-established practice to give indications on in vivo efficacy [57]. In this study, we used the K_p, uu_ values of eletriptan and sumatriptan determined herein in rats to estimate the unbound target-site concentrations in humans (Fig. 5A). These estimates were based on reported total maximal plasma concentrations (C_max_) of eletriptan and sumatriptan in patients [37, 38], adjusted for the f_u, plasma_ reported for humans [54]. Estimated unbound target-site concentrations were in combination with publicly available 5-HT_1B_, 5-HT_1D_, and 5-HT_1F_ receptor affinity constants used to predict therapeutic receptor occupancies in the trigeminal ganglion and in the whole brain according to Eq. 16 [55].

The receptor occupancy estimates as function of unbound plasma concentrations are shown in Fig. 5B-G. The substantial difference in the K_p, uu_ between the trigeminal ganglion and the whole brain was found to impact the regional receptor occupancy levels at clinically relevant plasma concentrations. We estimated almost complete 5-HT_1B/1D_ receptor occupancies in the trigeminal ganglion for both eletriptan and sumatriptan, while lower occupancies were estimated in the brain (Table 2). For example, at therapeutic C_max_, eletriptan was predicted to achieve 84% occupancy of 5-HT_1B_ receptors in the trigeminal ganglion and 37% in the brain. In contrast, the 5-HT_1F_ receptor occupancy for eletriptan was estimated to be considerably lower in both the whole brain and trigeminal ganglion.

Overall, our predictions indicate that both eletriptan and sumatriptan reach unbound concentrations that may be capable of stimulating 5-HT_1B/1D/1F_ receptors, not only in the trigeminal ganglion but also within the CNS at clinically relevant conditions.

## Discussion

The ability of triptans to cross the BBB sufficiently to contribute to their antimigraine effects via central 5-HT_1B/1D/1F_ receptors has been debated for decades [8, 9, 23, 27, 28]. Our study sought to clarify this by investigating the regional distribution of unbound eletriptan and sumatriptan in the PNS and CNS. We have shown that despite the low extent of BBB transport, eletriptan and sumatriptan may achieve unbound brain concentrations sufficient to engage with central targets, supporting the likelihood of an additional central site of action.

### Heterogenic triptan transport across PNS and CNS barriers with the most pronounced distribution into the trigeminal ganglion

Both eletriptan and sumatriptan demonstrated substantial differences in the extent of transport across the endothelial barriers of the PNS and CNS. The K_p, uu, whole brain_ values were ≤ 0.06 for both drugs indicating predominant active efflux at the BBB. Our findings align well with previously shown K_p, uu, brain_ values of 0.04 to 0.06 for eletriptan [20, 22, 58] and 0.05 to 0.07 for sumatriptan [58, 59]. Both eletriptan [19–22, 60] and sumatriptan [19, 61] have been identified as substrates of the efflux transporter, P-glycoprotein (P-gp). P-gp interactions may explain the low K_p, uu_ values across the BBB and BSCB. The efflux was preserved across all investigated brain regions, yet with extensively varying extents of transport (> 6-fold variation) indicating region-specific differences in BBB function. Inter-regional changes might influence the pharmacological potential of triptans depending on which regions are involved. The hypothalamus exhibited the highest brain exposure of both triptans. This increased exposure may be attributed to the median eminence, a circumventricular organ, in the hypothalamus, which might explain the higher K_p, uu_ found in this region [62].

We observed the highest drug distribution into the trigeminal ganglion with K_p, uu, TG_ values of 0.519 and 0.923 for eletriptan and sumatriptan, respectively. These findings indicate that sumatriptan freely penetrates the BNB via passive diffusion or mutually compensated efflux and influx, while eletriptan is subjected to moderate efflux. The higher triptan distribution into PNS regions might be a result of the BNB being less restrictive compared to the BBB, potentially due to a reduced influence of efflux transporters at the BNB [56]. In addition, a recent study demonstrated substantial differences in paracellular transport between the sciatic nerve barrier and the BBB, as indicated by differences in the K_p, uu_ of 4 kDa dextran [18]. This supports the notion that the BNB is a less restrictive barrier compared to the BBB.

### Predicted receptor occupancies in the CNS and potential clinical implications

Despite limited BBB penetration of eletriptan and sumatriptan leading to low unbound concentrations in CNS regions, our predictions indicate that central targets may still be relevant at therapeutic doses (Table 2). Notably, eletriptan is estimated to reach a 5-HT_1D_ receptor occupancy of 67% in the whole brain at its peak plasma concentration. This relatively high receptor occupancy in the CNS emphasizes the potential clinical implication herein. In contrast, eletriptan is associated with a considerably lower occupancy of 5-HT_1F_ receptors in the whole brain, estimated around 15%.

Using mathematical predictions, Tokuora et al. (2014) suggested that a 5-HT_1B_ receptor occupancy of 32.0-89.4% and a 5-HT_1D_ receptor occupancy of 68.4–96.2% are necessary to reach clinical effects [54]. These estimates were based on unbound plasma concentrations associated with clinical efficacy, however, not accounting for unbound target-site concentrations [54]. Based on our predictions of brain receptor occupancy, eletriptan is likely to achieve the lower threshold proposed by Tokuora et al. for both receptors at its peak plasma concentration following oral administration of 40 mg. In contrast, sumatriptan is expected to reach the minimally required receptor occupancy only for the central 5-HT_1B_ receptors after a 100 mg oral dose (Table 2). While the exact receptor occupancy required for the clinical effects of triptans remains unclear, some suggest that even lower receptor occupancy levels might be sufficient for the clinical efficacy of sumatriptan [63]. A positron emission tomography (PET) study showed that sumatriptan displaces a 5-HT_1B_ receptor ligand with a mean occupancy rate of 16 ± 5.3% after clinically relevant dosing of 6 mg subcutaneous injection, reflecting a C_tot, max_ of 72 ng/mL, to a patient experiencing migraine [63, 64]. The mean 16% receptor occupancy of 5-HT_1B_ receptors was demonstrated in the seven pain-modulating regions including the dorsolateral and ventrolateral prefrontal cortex, orbitofrontal cortex, anterior cingulate cortex, sensorimotor cortex, insula, and amygdala [63]. In this regard, our estimates of 21–32% central 5-HT_1B_ receptor occupancy in the whole brain for sumatriptan at 30 ng/mL and 53 ng/mL peak plasma concentrations are in line with the PET imaging study of sumatriptan. It is important to note that the frontal and parietal cortices exhibited the lowest extent of BBB transport in healthy rats, and, on average, unbound ISF concentrations in these regions are expected to be approximately two-fold lower than the whole-brain concentrations used for receptor occupancy estimation. Despite this, our estimates indicate that sumatriptan at doses of 100 mg or higher can achieve 15% or greater receptor occupancy of the 5-HT_1B_ receptor in the cortical areas.

Reported CNS-related side effects after triptan administration support the prospect of central receptor engagement [25]. Ferrari et al. (2002) reported placebo-subtracted CNS side effects of 3.7% after peroral administration of 50 mg sumatriptan, while patients receiving 40 mg eletriptan demonstrated placebo-subtracted CNS side effects of 7.5% [25, 26]. The higher incidence of CNS-related side effects after eletriptan administration complies well with the present study indicating that eletriptan is more likely to stimulate central receptors as compared to sumatriptan (Table 2).

Although we have not investigated the antimigraine effect, herein we demonstrate that a central effect exerted by eletriptan and sumatriptan cannot be excluded, despite their poor BBB penetrating properties.

### Study limitations – evaluation of the assumptions

Several assumptions underlie the predictions of receptor occupancies in humans based on rat K_p, uu_ and human in vitro affinity values, each carrying potential limitations that must be acknowledged. One key assumption is that K_p, uu_ is concentration independent. To obtain full concentration-receptor occupancy profiles, simulations were performed within the unbound plasma concentration range of 0.1–10,000 nM. Although concentration-dependent changes in K_p, uu_ could theoretically occur upon saturation of transporters, this is unlikely for P-gp substrates [65, 66]. Moreover, the upper concentration range is unlikely to be achieved at therapeutic doses, making this assumption well-founded for therapeutic predictions. Species differences in the BBB are another critical consideration. As noted previously, both eletriptan [19–22, 60] and sumatriptan [19, 61] are substrates of P-gp, an efflux transporter that is expressed in brain microvasculature at higher levels in rats compared to humans. Uchida et al. (2020) reported that the P-gp expression at the BBB is 10-fold greater in rats than in humans [67]. Consequently, the K_p, uu, brain_ of P-gp substrates, such as eletriptan and sumatriptan, may be higher in humans than in rats. Bauer et al. examined (R)-[^11^C]verapamil, a known P-gp substrate, in the presence of increasing doses of the P-gp inhibitor tariquidar in a PET imaging study. They showed a 4-fold more pronounced response to P-gp inhibition in rats compared to humans [66]. The latter indicates a potential discrepancy between the absolute protein content and its function. Since the quantitative assessment of K_p, uu, brain_ in humans remains to be one of the ground challenges in the BBB field, we are unable to provide accurate estimates of the level of potential underestimation of brain exposure for the investigated triptans.

In addition, we assumed consistent 5-HT_1B/1D/1F_ expression levels across the studied regions. However, conflicting data may weaken this assumption. On one hand, variations in 5-HT_1B/1D/1F_ receptor expression levels across various brain regions have been reported, wherefore our results must be interpreted with caution [13, 15]. Variability in receptor expression could influence the regional receptor occupancies of triptans. On the other hand, Deen et al. (2019) reported that sumatriptan might occupy 5-HT_1B_ receptors uniformly across various brain regions, suggesting that the selection of specific regions for analysis is unlikely to lead to misinterpretation [63].

Some triptans, such as eletriptan, are known to have active metabolites [26]. In the present study, only parent compounds were analyzed. Since metabolites may have a clinical impact, this is a potential limitation of the study.

In conclusion, while these assumptions are well-founded, they introduce uncertainties that must be considered when interpreting the study findings. If assumptions are violated, it is more likely to result in underestimation rather than overestimation, strengthening the hypothesis of the central site of action.

## Conclusion

This study provides the first comprehensive evaluation of eletriptan and sumatriptan distribution across various regions of the CNS and PNS, offering insights into triptans’ potential target-sites in migraine therapy. The highest triptan exposure was observed in the trigeminal ganglion, which is a well-known antimigraine target-site. In contrast, both eletriptan and sumatriptan displayed limited BBB transport, with notable inter-regional variations. We predicted almost complete receptor occupancy in the trigeminal ganglion, with markedly lower but still notable occupancies in the brain, regardless of triptan and receptor subtype.

In conclusion, these findings suggest that eletriptan and sumatriptan can engage with central receptors despite limited BBB transport, supporting their potential for both peripheral and central sites of action in migraine therapy. Although eletriptan and sumatriptan may engage with central receptors, the impact of a central action is still uncertain. Further research is needed to explore whether central receptor occupancy contributes to antimigraine effects and/or CNS side effects in patients.

## Supplementary Information

Additional file 1: The bioanalytical method parameters and sample preparation details. Tabel S1. The MS/MS parameter employed for the bioanalysis of sumatriptan, sumatriptan-d6 and eletriptan. Table S2. Liquid chromatography conditions used for bioanalysis of eletriptan and sumatriptan

Additional file 2: Time-concentration plasma profiles and endpoint concentration of eletriptan and sumatriptan. Plasma concentrations of eletriptan and sumatriptan after a 4-hour IV infusion in rats. A) Time-concentration profiles of eletriptan and sumatriptan after a 4-hour IV infusion in male rats. Data not showed for females due to a technical error. B) Endpoint plasma concentrations after terminal heart puncture in male and female rats. Each data point or column represents the mean ± SD.

Additional file 3: K_p_ and K_p,uu_ with linear scale. Assessment of regional K_p_ and K_p,uu_ for eletriptan and sumatriptan in rats under steady state. A) Total tissue-to-plasma concentration ratio. B) The unbound tissue-to-plasma concentration ratio. Columns represent mean ± SD. The mean value of each column is annotated within each bar. The dotted line represents the line of unity. Values below unity indicate predominant active efflux across the respective barriers. Values are sorted according to descending K_p_/K_p,uu_ values for eletriptan.

Additional file 4: Unbound fractions of eletriptan and sumatriptan in plasma. Unbound fractions of eletriptan and sumatriptan in plasma assessed at 200 nM and 400 nM. Each column represents the mean ± SD. Groups were compared using a two-way ANOVA analysis followed by a Tukey’s multiple comparison test

Additional file 5: Unbound fractions of eletriptan and sumatriptan in brain and nerve homogenate. Unbound fractions of eletriptan and sumatriptan in brain and sciatic nerve homogenate. Each column represents the mean ± SD.

## Data Availability

No datasets were generated or analysed during the current study.

## Abbreviations

5-HT_1B_: 5-hydroxytryptamine receptor subtype 1B
5-HT_1D_: 5-hydroxytryptamine receptor subtype 1D
5-HT_1F_: 5-hydroxytryptamine receptor subtype 1F
aECF: Artificial extracerebral fluid
BBB: Blood-brain barrier
BNB: Blood-nerve barrier
BSCB: Blood-spinal cord barrier
CaCl_2_: Calcium dichloride
CMA: Combinatory mapping approach
C_max_: Peak plasma concentration
CNS: Central nervous system
f_u,brain_: Fraction unbound in brain
f_u,nerve_: Fraction unbound in nerve
f_u,plasma_: Fraction unbound in plasma
HEPES: N’-2-Hydroxyethylpiperazine-N’-2 ethane sulphonic acid
ICF: Intracellular fluid
ISF: Interstitial fluid
IV: Intravenous
KCl: Potassium chloride
K_d_: Equilibrium dissociation constants
KH_2_PO_4_: Potassium dihydrogen phosphate
K_p_: Total tissue-to plasma concentration ratio
K_p,uu,cell_: Intracellular-to-extracellular cell partitioning coefficient
K_p,uu_: Unbound tissue-to-plasma concentration ratio
LLOQ: Lower limit of quantification
MgSO_4_: magnesium sulfate
MPA: Mobile phase A
MPB: Mobile phase B
NaCl: sodium chloride
NeuroPK: Neuropharmacokinetic
NS: Nervous system
PBS: Phosphate-buffered saline
PET: Positron emission tomography
PNS: Peripheral nervous system
PO: Peroral
QC: Quality control
RO: Receptor occupancy
ROI: Region of interest
Rpm: Rotations per minute
Ss: Steady state
TG: Trigeminal ganglion
ULOQ: Upper limit of detection
UPLC-MS/MS: Ultra-performance liquid chromatography coupled with a tandem mass spectrometry
v:v: Volume: volume
V_eff_: Effective plasma volume
V_protein_: Brain vascular volume of plasma protein
V_u,brain_: Unbound volume unbound in brain
V_water_: Brain vascular volume of plasma water
w:v: Weigh: volume
WB: Whole brain
