# Supplementary material for: Regional distribution of unbound eletriptan and sumatriptan in the CNS and PNS in rats: implications for a potential central action
Source: J Headache Pain. 2024 Oct 30;25(1):187. doi: 10.1186/s10194-024-01894-0 (PMC11523665; doi:10.1186/s10194-024-01894-0)
Supplement: Supplementary file 1 — Additional file 1: The bioanalytical method parameters and sample preparation details. Tabel S1. The MS/MS parameter employed for the bioanalysis of sumatriptan, sumatriptan-d6 and eletriptan. Table S2. Liquid chromatography conditions used for bioanalysis of eletriptan and sumatriptan [file 10194_2024_1894_MOESM1_ESM.docx]

## Additional file 1: The bioanalytical method parameters and sample preparation details

### Tabel S1. The MS/MS parameter employed for the bioanalysis of sumatriptan, sumatriptan-d6 and eletriptan.

| Compound | Precursor-product ion pair  (m/z) | Cone voltage  (V) | Collision energy  (V) |
| --- | --- | --- | --- |
| Sumatriptan | 296.20 🡪 58.00 | 40 | 15 |
| Sumatriptan-d6 | 301.60 🡪 64.00 | 40 | 15 |
| Eletriptan | 383.20 🡪 84.00 | 40 | 25 |

The analysis was performed in positive ion mode, with a source temperature of 150°C and desolvation temperature of 500°C.

### Table S2. Liquid chromatography conditions used for bioanalysis of eletriptan and sumatriptan.

| Time  (min) | Injection volume  (µL) | Flow rate  (mL/min) | Percentage of MPA  (%) | Percentage of MPB  (%) |
| --- | --- | --- | --- | --- |
| Initial | 5 | 0.3 | 95.0 | 5.0 |
| 0.30 | 5 | 0.3 | 95.0 | 5.0 |
| 1.80 | 5 | 0.3 | 5.0 | 95.0 |
| 2.20 | 5 | 0.3 | 5.0 | 95.0 |
| 2.25 | 5 | 0.3 | 50.0 | 50.0 |
| 2.50 | 5 | 0.3 | 50.0 | 50.0 |
| 3.00 | 5 | 0.3 | 5.0 | 95.0 |
| 3.30 | 5 | 0.3 | 5.0 | 95.0 |
| 3.40 | 5 | 0.3 | 95.0 | 5.0 |
| 3.50 | 5 | 0.3 | 95.0 | 5.0 |

Sample manager temperature: 4°C, column temperature: 40°C, MPA: mobile phase A, MPB: mobile phase B. The retention times for sumatriptan, sumatriptan-d6 and eletriptan were found to be 2.16 min, 1.88 min, and 1.87 min, respectively. The total run time was 4.0 min.

### Matrix effect and carryover

Matrix matched external standard and coeluting internal standard method had been selected for sumatriptan and eletriptan peaks quantification. Respective blank matrix samples with and without internal standard were included in the analytical runs to confirm the absence of contamination and carryover. Different organic solvents and proportions were checked to optimize protein precipitation. Optimal dilution proportions had been chosen for simultaneous quantification of sumatriptan and eletriptan concentrations (refer, Sample preparation section). Carryover was assessed by analyzing blank samples after the calibration standard at the upper limit of quantification (ULOQ). To avoid any potential impact of carryover on the samples, blank samples or MPA were included after ULOQ standards, samples with an expected high concentration, and before the next study sample (refer, Acceptance criteria for validation section). Concentration ranges for all samples were higher than lower limit of quantification (LLOQ) and could not be affected by carryover.

Standards and quality control (QC) samples were prepared in blank plasma and brain homogenate. Blank brains from three drug-naïve rats were homogenized 1:4 (w:v) with Milli-Q water, 1:9 (w:v) with aECF, and 1:19 (w:v) with PBS, pH 7.4 for the neuroPK, equilibrium dialysis and rat brain slice assay experiments, respectively. The brains were homogenized on ice by ultrasonication for three cycles (5 s “on” and 5 s “off”) at an amplitude of 50 % using an ultrasonic processor VCX-130 (Sonics, Chemical Instruments AB, Sweden). Ready to use brain homogenate is kept at 4 °C.

### Standard and quality control (QC) samples

Non-zero calibration ranges for neuroPK analysis were from 0.1 ng/mL to 220 ng/mL for sumatriptan and eletriptan in blank plasma and brain homogenate:Milli-Q water 1:4 (w:v) (12 levels). Five levels of QC samples for both matrices were prepared with concentrations of sumatriptan and eletriptan of 0.8, 4.0, 40, 80, and 120 ng/mL. Standards with 0.5 nM to 1000 nM (12 levels) concentrations of sumatriptan and eletriptan in blank plasma, brain homogenate with Milli-Q water, 1:9 (w:v) with aECF, and 1:19 (w:v) with PBS, pH 7.4 solution, were prepared to ensure calibration curve linearity check for brain slice assay and equilibrium dialysis experiments, respectively.

### Calibration curves

The calibration curves were constructed using linear regression and a weighing function of 1/x^2^, which resulted in an even residual distribution and similar importance for all the concentrations in the calibration range. The linear range with a determination coefficient (R^2^) equal to or higher than 0.99 was obtained in all standard curves prepared in the respective control matrices using the cassette approach, i.e., including sumatriptan and eletriptan at each standard level. Respective blank matrix samples with and without internal standard were included in the analytical runs to confirm the absence of contamination and carry-over. The concentration range in the standard curves was 0.5-220 ng/mL for both sumatriptan and eletriptan (10 levels) in plasma and blank brain homogenate, and five levels QC samples for both matrices were prepared with concentrations of sumatriptan and eletriptan of 0.8, 4.0, 40, 80, and 120 ng/mL. 2-1000 nM (8 levels) for the equilibrium dialysis and brain slice assay experiments, respectively. The lowest standard points were set as LLOQ in both matrices.

### Sample preparation

Protein precipitation was performed on plasma and tissue samples, respective standards, QC samples, and blanks plasma and tissues in batches of 12-16 samples or 96 well format. All preparations were performed protected from light maintaining at 4 ˚C to give all samples the same time aspect and minimize the risk of degradation, evaporation, and pellet dissociation. Sample preparation was conducted in two steps. Plasma and tissue homogenate samples, with respective blanks, standards and quality controls were precipitated in acetonitrile 1:3 (v:v) with internal standard (sumatriptan-d6) followed by centrifugation (3 min, 13.000 rpm). The supernatant was diluted in MPA 1:2 (v:v).

Briefly, samples, standards, QCs, and blanks were thawed and vortexed for 2 minutes. A sample volume of 50 µL was precipitated with 150 µL acetonitrile containing 20 ng/mL sumatriptan-d6 internal standard using 1.5 mL micro tubes (Sarstedt, Numbrecht, Germany) or Costar v-bottom 0.5 mL 96-well plate (Corning Inc., Salt Lake City, USA) covered with adhesive aluminum foil for microplates (VWR, Sweden) and vortexed for 1 minute for plasma and 5 minutes for tissue samples. After vortexing, samples were centrifuged for 5 minutes at 4100 rpm at 4 ˚C. 50 µL of supernatant was transferred to 1.1 mL short thread HPLC vials with inner cone (VWR, Sweden) or in Costar round bottom 1 mL 96-well plate (Corning Inc., NY, USA), containing 100 µL MPA. Solutions were vortexed up to 2 minutes and placed into an autosampler (4 °C). The sample volume injected onto the column was 5 µL.

### Stability

Stability of sumatriptan and eletriptan up to 6 h at bench top, 48 h in an autosampler at 4 °C, up to 3 months at - 20 °C, 3 months at - 80 °C and after three freeze–thaw cycles at - 20 °C both alone and in the presence of eletriptan/sumatriptan in different matrices were assessed (40-45, 48). In this study, 1 day matrix stability experiments were conducted to cover the laboratory handling conditions. All results reported are obtained from samples stored at - 20 °C and assessed within one month from receival using freshly thawed standards and QCs protected from light and maintained at 4 °C.

### Acceptance criteria for an analytical run

Several acceptance criteria were predefined for sumatriptan and eletriptan quantification in plasma and tissue samples (49). Calibration standards used were freshly prepared in the same biological matrix as the study samples. The linear range with a determination coefficient (R^2^) was equal to or higher than 0.99. The back-calculated concentrations of the calibration standards should be within ± 15 % of the nominal value, except for the LLOQ, for which ± 20 % deviation is accepted. At least 75 % of the calibration standards, including a minimum of six concentration levels, should fulfill these criteria. At least 2/3 of the total QCs and at least 50 % at each concentration level should be within ± 15 % of the nominal values. The injections of blank samples should be included after the ULOQ standards, samples with an expected high concentration, and before the next study sample to eliminate the impact of potential carryover.
