# Supplementary material for: Regional distribution of unbound eletriptan and sumatriptan in the CNS and PNS in rats: implications for a potential central action
Source: J Headache Pain. 2024 Oct 30;25(1):187. doi: 10.1186/s10194-024-01894-0 (PMC11523665; doi:10.1186/s10194-024-01894-0)
Supplement: Supplementary file 2 — Additional file 2: Time-concentration plasma profiles and endpoint concentration of eletriptan and sumatriptan. Plasma concentrations of eletriptan and sumatriptan after a 4-hour IV infusion in rats. A) Time-concentration profiles of eletriptan and sumatriptan after a 4-hour IV infusion in male rats. Data not showed for females due to a technical error. B) Endpoint plasma concentrations after terminal heart puncture in male and female rats. Each data point or column represents the mean ± SD. [file 10194_2024_1894_MOESM2_ESM.docx]

## Additional file 2: Time-concentration plasma profiles and endpoint concentration of eletriptan and sumatriptan


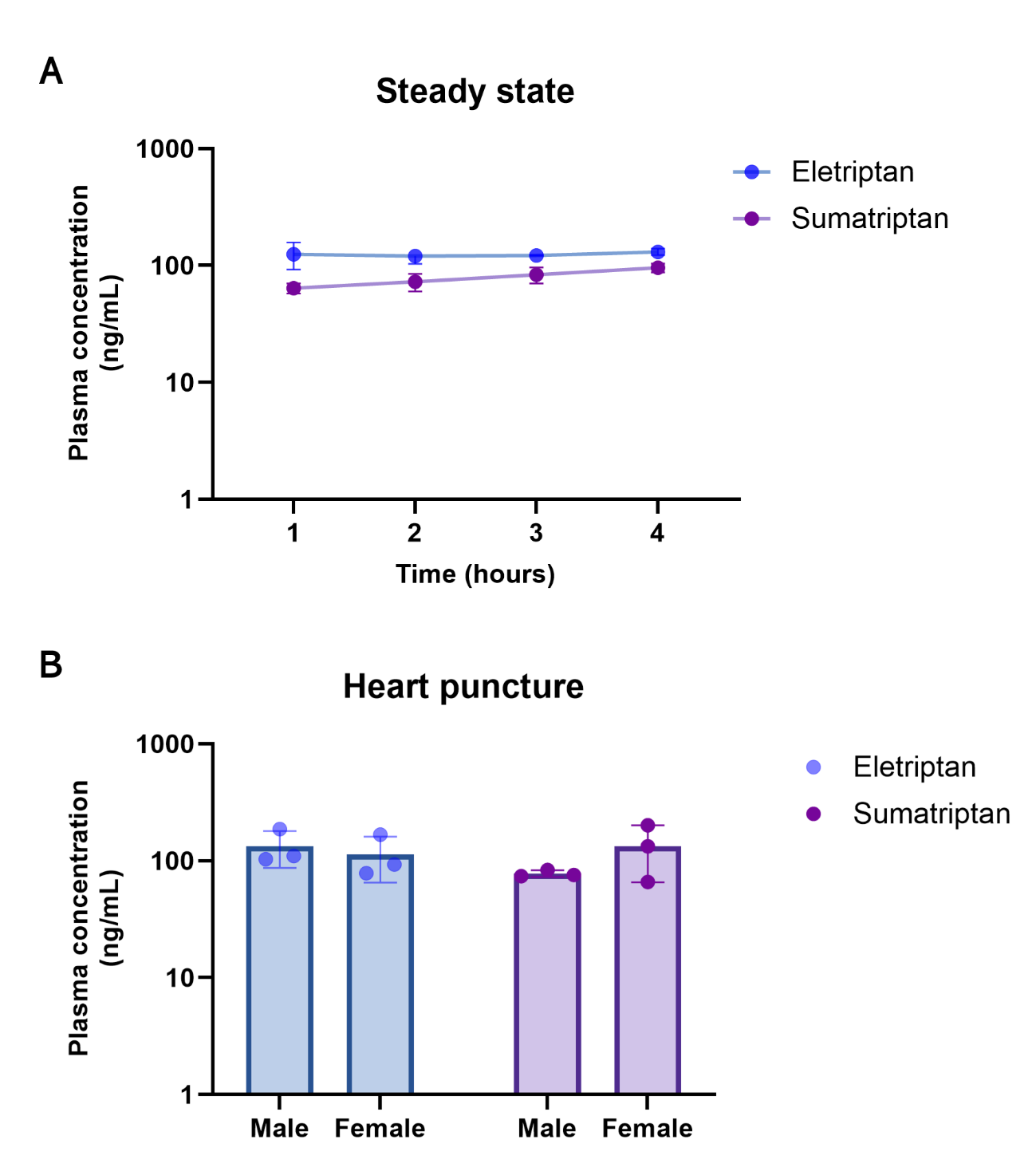


**Additional file 2. Plasma concentrations of eletriptan and sumatriptan after a 4-hour IV infusion in rats. A)** Time-concentration profiles of eletriptan and sumatriptan after a 4-hour IV infusion in male rats. Data not showed for females due to a technical error. **B)** Endpoint plasma concentrations after terminal heart puncture in male and female rats. Each data point or column represents the mean ± SD (N=3)$.$
