# Supplementary material for: Regional distribution of unbound eletriptan and sumatriptan in the CNS and PNS in rats: implications for a potential central action
Source: J Headache Pain. 2024 Oct 30;25(1):187. doi: 10.1186/s10194-024-01894-0 (PMC11523665; doi:10.1186/s10194-024-01894-0)
Supplement: Supplementary file 3 — Additional file 3: Kp and Kp,uu with linear scale. Assessment of regional Kp and Kp,uu for eletriptan and sumatriptan in rats under steady state. A) Total tissue-to-plasma concentration ratio. B) The unbound tissue-to-plasma concentration ratio. Columns represent mean ± SD. The mean value of each column is annotated within each bar. The dotted line represents the line of unity. Values below unity indicate predominant active efflux across the respective barriers. Values are sorted according to descending Kp/Kp,uu values for eletriptan. [file 10194_2024_1894_MOESM3_ESM.docx]

## Additional file 3: K_p_ and K_p,uu_ with linear scale


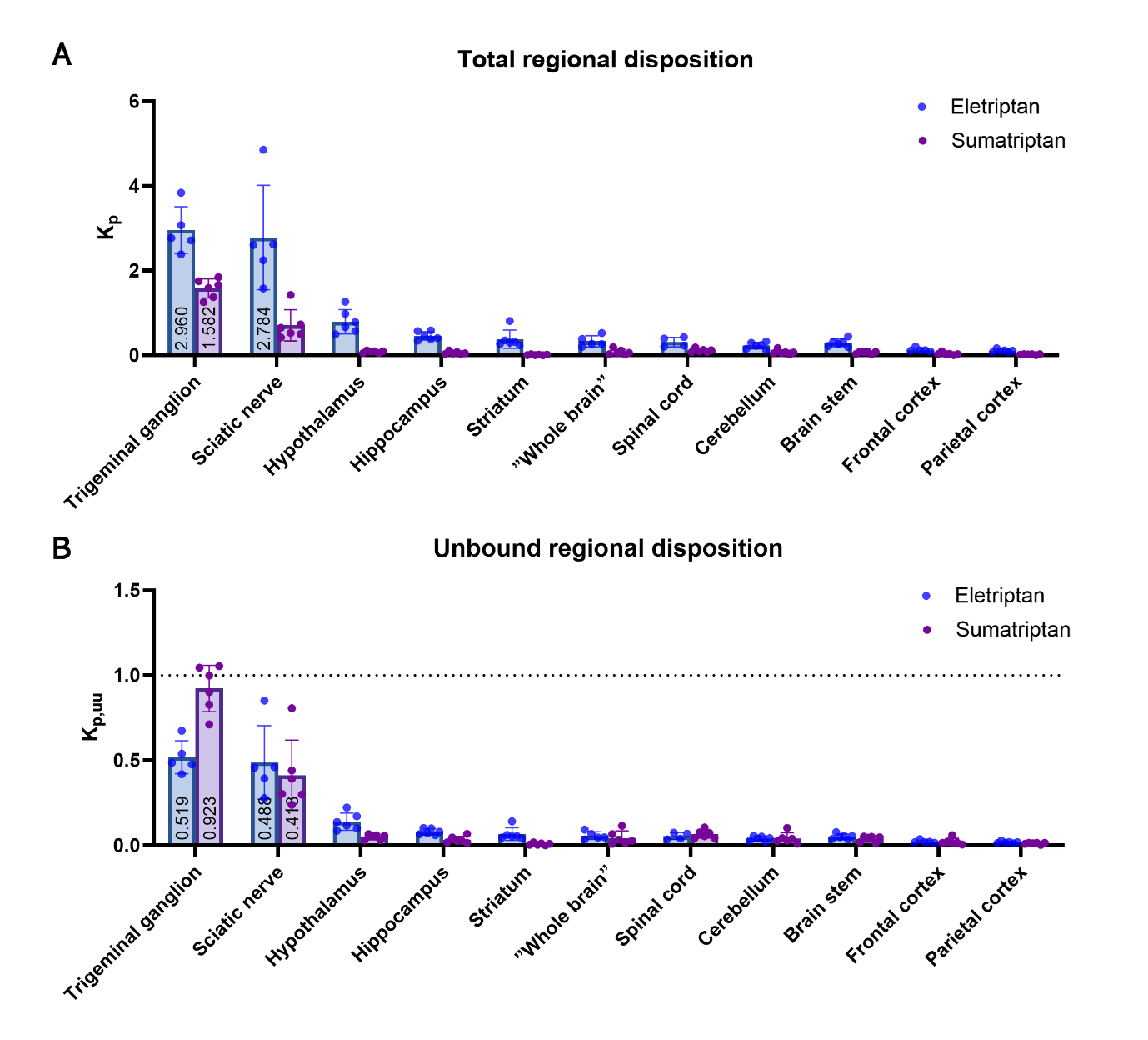


**Additional file 3. Assessment of regional K_p_ and K_p,uu_ for eletriptan and sumatriptan in rats under steady state. A)** Total tissue-to-plasma concentration ratio (K_p_). **B)** The unbound tissue-to-plasma concentration ratio (K_p,uu_). Columns represent mean ± SD (n= 4-6). The mean value of each column is annotated within each bar. The dotted line represents the line of unity. Values below unity indicate predominant active efflux across the respective barriers. In Fig. 3B and 3C, regions are sorted according to descending K_p_/K_p,uu_ values for eletriptan
