# Supplementary material for: Regional distribution of unbound eletriptan and sumatriptan in the CNS and PNS in rats: implications for a potential central action
Source: J Headache Pain. 2024 Oct 30;25(1):187. doi: 10.1186/s10194-024-01894-0 (PMC11523665; doi:10.1186/s10194-024-01894-0)
Supplement: Supplementary file 4 — Additional file 4: Unbound fractions of eletriptan and sumatriptan in plasma. Unbound fractions of eletriptan and sumatriptan in plasma assessed at 200 nM and 400 nM. Each column represents the mean ± SD. Groups were compared using a two-way ANOVA analysis followed by a Tukey’s multiple comparison test [file 10194_2024_1894_MOESM4_ESM.docx]

## Additional file 4: Unbound fractions of eletriptan and sumatriptan in plasma


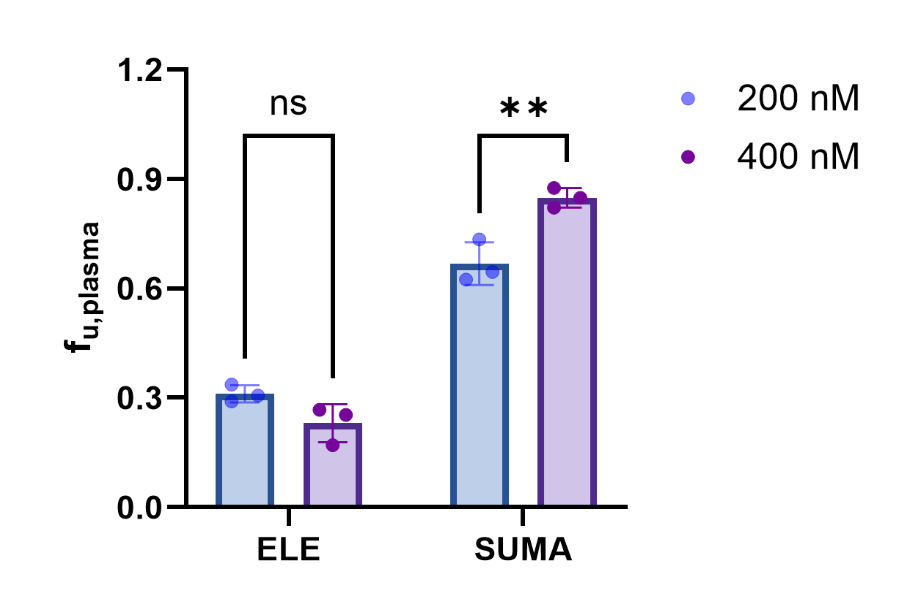


**Additional file 4. Unbound fractions of eletriptan and sumatriptan in plasma assessed at 200 nM and 400 nM.** Each column represents the mean ± SD (n= 3, N= 3). Groups were compared using a two-way ANOVA analysis followed by a Tukey’s multiple comparison test.
