# Supplementary material for: Regional distribution of unbound eletriptan and sumatriptan in the CNS and PNS in rats: implications for a potential central action
Source: J Headache Pain. 2024 Oct 30;25(1):187. doi: 10.1186/s10194-024-01894-0 (PMC11523665; doi:10.1186/s10194-024-01894-0)
Supplement: Supplementary file 5 — Additional file 5: Unbound fractions of eletriptan and sumatriptan in brain and nerve homogenate. Unbound fractions of eletriptan and sumatriptan in brain and sciatic nerve homogenate. Each column represents the mean ± SD. [file 10194_2024_1894_MOESM5_ESM.docx]

## Additional file 5: Unbound fractions of eletriptan and sumatriptan in brain and nerve homogenate


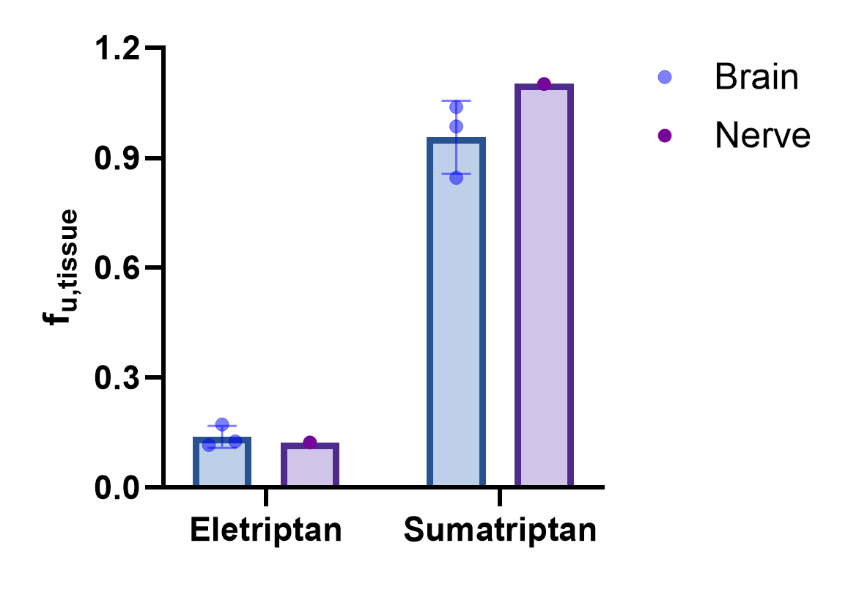


**Additional file 5. Unbound fractions of eletriptan and sumatriptan (1 µM) in brain and sciatic nerve homogenate.** Each column represents the mean ± SD. Brain (n= 3, N= 3), sciatic nerve (n=1, N= 2).
